# How and Why Men and Women Differ in Their Microbiomes: Medical Ecology and Network Analyses of the Microgenderome

**DOI:** 10.1002/advs.201902054

**Published:** 2019-10-23

**Authors:** Zhanshan (Sam) Ma, Wendy Li

**Affiliations:** ^1^ Computational Biology and Medical Ecology Lab State Key Laboratory of Genetic Resources and Evolution Kunming Institute of Zoology Chinese Academy of Sciences Kunming 650223 China; ^2^ Center for Excellence in Animal Evolution and Genetics Chinese Academy of Sciences Kunming 650223 China

**Keywords:** autoimmune diseases (AD), core/periphery network (CPN), high‐salience skeleton network (HSN), human microbiome associated diseases, microgenderome

## Abstract

Microgenderome or sexual dimorphism in microbiome refers to the bidirectional interactions between microbiotas, sex hormones, and immune systems, and it is highly relevant to disease susceptibility. A critical step in exploring microgenderome is to dissect the sex differences in key community ecology properties, which has not been systematically analyzed. This study aims at filling the gap by reanalyzing the Human Microbiome Project datasets with two objectives: (i) dissecting the sex differences in community diversity and their intersubject scaling, species composition, core/periphery species, and high‐salience skeletons (species interactions); (ii) offering mechanistic interpretations for (i). Conceptually, the Vellend–Hanson synthesis of community ecology that stipulates selection, drift, speciation, and dispersal as the four processes driving community dynamics is followed. Methodologically, seven approaches reflecting the state‐of‐the‐art research in medical ecology of human microbiomes are harnessed to achieve the objectives. It is postulated that the revealed microgenderome characteristics (categorized as seven aspects of differences/similarities) exert far reaching influences on disease susceptibility, and are primarily due to the sex difference in selection effects (deterministic fitness differences in microbial species and/or species interactions with each other or with their hosts), which are, in turn, shaped/modulated by host physiology (immunity, hormones, gut–brain communications, etc.).

## Graphical abstract

The microgenderome (sexual dimorphism in microbiome) refers to bidirectional interactions between microbiotas, sex hormones, and immune systems, and it is highly relevant to disease susceptibility. Medical ecology and network analyses reveal seven microgenderome characteristics, that is, sex differences in community diversity, intersubject scalings of heterogeneity and diversity, shared (unique) species, special trio motifs and modules, core/periphery, and high‐salience skeletons.

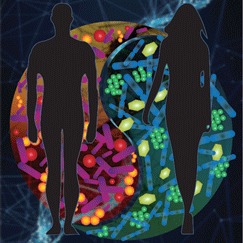

## 1 Introduction

The sex difference or sexual dimorphism in immunity (particularly autoimmunity) is influenced by gut microbiota.^1^, ^2^, ^3^ Sex differences in gut microbiome are partially driven by sex hormones, which in turn contribute to sex differences in immunity and susceptibility to a multitude of infections and chronic diseases.^4^, ^5^, ^6^, ^7^, ^8^, ^9^ The microgenderome defines the interaction between microbiota, sex hormones, and the immune system, and it involves bidirectional interactions between the microbiota, hormones, immunity, and disease susceptibility.^5^, ^6^

A major significance for microgenderome research is its high relevance to disease susceptibility. Emerging evidence suggests that sex‐associated differences in gut microbiota can influence sex‐specific susceptibility to disease. Man and woman can differ in the nature and strength of immune responses, leading to sex‐specific differences in the prevalence, manifestations, and outcomes of malignancies, autoimmune and infectious diseases.^10^ Particularly, sex bias is an important aspect of many autoimmune diseases (ADs). Studies have discovered a bidirectional cross talk between microbiota and the endocrine system, in which bacteria are able to produce hormones, respond to host hormones, and regulate host hormones' homeostasis through inhibiting gene transcription. In turn, host hormones may influence bacterial gene expression, bacterial virulence and growth, with consequences on host physiology.^7^ A better understanding of the fundamental processes that regulate sex‐specific differences in immune responses in the context of microgenderome is required to optimize prevention and treatment strategies for women and men as a first step toward personalized precision medicine.^10^

Microbiome may act as coach for immune system or as amplifier of autoimmunity.^11^, ^12^ The early development of the immune system may be modulated by microbial colonization in mucosal tissues.^11^ Within an individual's life span, host constantly shapes the gut microbiome through the immune system.^12^ Microgenderome may also play an important role in gut–brain–axis communication; the bidirectional communication between the gut microbiome and the brain has been considered as a factor that influences immunity, metabolism, neurodevelopment, and behavior.^13^

In summary, as in all other fields of biomedicine, there is an undeniable need to explore microgenderome.^14^ While the interaction between microbiota, sex hormones, and the immune system is certainly of critical significance as emphasized previously, a comprehensive ecological analysis, particularly from species interaction or network perspective should be of equal importance, but still missing in existing literature to the best of our knowledge. We fill this gap in the present study by leveraging some recent methodological advances in medical ecology and network science.^15^, ^16^, ^17^ Specifically, we perform the following seven ecological/network analyses by reanalyzing the human microbiome project (HMP) datasets,^18^ including: (i) community diversity comparisons based on the Hill numbers,^19^ (ii) shared species analysis based on the study of Ma et al.;^20^ (iii) intersubject heterogeneity comparisons based on extended power law analysis;^21^ (iv) diversity‐scaling analysis based on diversity–area relationship (DAR);^22^, ^23^ (v) basic species co‐occurrence network (SCN);^24^, ^25^, ^26^ (vi) shared core/periphery network (CPN);^15^, ^27^ (vii) shared high‐salience skeleton network (HSN).^15^, ^28^ While the first four analyses focus on the species diversity and composition, the last three focus on species interactions. The basic network analysis is aimed to compare network properties and motifs. The shared CPN and HSN analyses compare the core nodes (species) and critical skeletons (the backbone of species interactions) between both sexes, respectively.

## 2 Results and Discussion

Next, we summarize our findings and their implications as seven aspects by following the seven methods introduced in the Experimental Section.

### 2.1 Sex Differences in Microbiome Diversity with Hill Numbers

We performed the comparisons in species diversity between both the sexes with Hill numbers at three layers. First, the comparison was conducted at the whole community level by computing and comparing the Hill numbers with all species in the community sample. Second, the comparison was conducted for five major phyla, respectively, including *Actinobacteria, Bacteroidetes, Firmicutes, Fusobacteria*, and *Proteobacteria*. Third, the comparison was conducted by distinguishing species as *core* species and *periphery* species, which is supported by the CPN analysis.

At the whole community level, 18 out of 60 (15 sites and 4 diversity orders) or 30% possible comparisons between the male and female exhibited statistically significant differences. In terms of the sites, 7 out of 15 sites exhibited significant sex differences. Among the seven sites with differences (*M* ≠ *F*), in two oral sites (palatine tonsils, saliva) sites, *F* > *M*, and in the remaining five sites (4 skin sites and anterior nares) the *M > F*. In 8 out of possible 15 sites, including gut microbiome, no significant sex differences (*M* ≈ *F*) were detected.

**Figure 1:**
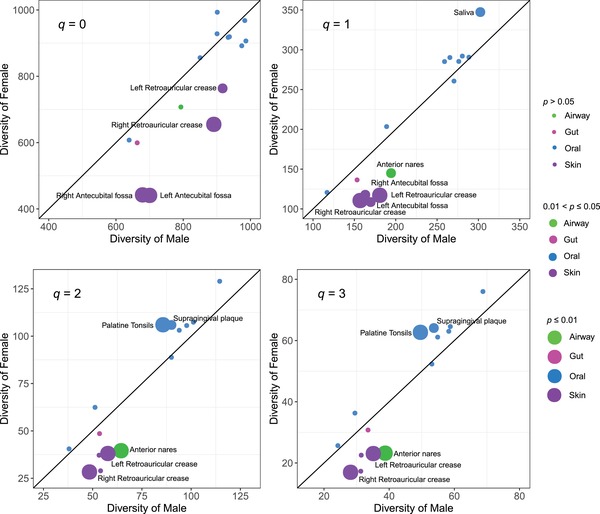
Comparison of species diversity in Hill numbers (at *q* = 0–3) of the male versus female: Solid circles with different color represent for different microbiome sites (i.e., green = airway, magenta = gut, blue = oral, purple = skin). Circle size represents for the size of *p*‐value from Wilcoxon test; the greater the diversity difference, the smaller the *p*‐value, and the larger the circle size is accordingly. The farther from the 45° line (equal diversity line of the male vs female), and the larger the diversity difference between the male and female. See Table S1–1 (Supporting Information) for the detailed information of the diversity comparisons.

**Figure** **1** (Wilcoxon test), Figure S1 (Supporting Information) (*d*‐statistic for effect size), and Tables S1‐1 (Supporting Information) displayed the results of comparing the community‐level diversity between the male and female at each of the 15 microbiome sites. Here, we only note the sites that exhibited significant sex differences. At diversity orders *q* = 0 and *q* = 1, at all four skin sites, *M > F*. At *q* = 1, at anterior nares (airway microbiome), *M* > *F*. Also at *q* = 1, *M* < *F* for *saliva* microbiome. At *q* = 2 and 3, only four sites including left retroauricular crease, right retroauricular crease, anterior nares, and palatine tonsils, *M ≠ F*, and in other 11 sites including gut microbiome, *M* ≈ *F*.

We further dissect the sex differences by focusing on the five major phyla (see the legends for Figures S2 and Figure S3, and Table S1‐2, Supporting Information, for the detailed discussion). Two points are particularly worthy of reiterating here: (i) When we look into the phylum level, the species diversity (at diversity order *q* = 0 and 1) of *Bacteroidetes* in the gut was *M > F*, although the overall community‐level diversity of gut was *M* ≈ *F*. Interestingly, *Firmicutes*, another key phylum, was *M* ≈ *F* in the gut microbiome, but was *M < F* in the saliva microbiome. (ii) *Actinobacteria* and *Firmicutes* also play important roles in determining sex differences.

The third‐layer diversity analysis, comparing species diversity by distinguishing species as core and periphery species based on the CPN construction and analysis, suggested that the sex differences are more far reaching than what were revealed from the analyses conducted at the previous two layers (see Figures S4–S6, Table S1–3, Supporting Information). For example, for the gut (stool) microbiome, the species diversity of core‐species was *M > F* at diversity orders *q* = 1, 2, and 3, the diversity of periphery‐species was *M* > *F* at diversity order *q* = 0. This is again in contrast with the previous community‐level diversity comparisons, in which gut microbiome was *M* ≈ *F*.

### 2.2 Shared Species Analysis between the Male and Female

While the diversity analysis in the previous section reveals the sex‐specific characteristics from species diversity perspective, the shared species analysis in this section focuses on the species composition. When the number of shared species between both sexes exhibits no significant difference than that expected by chance, it indicates that the shared species between a pair of men, a pair of women, or a pair of man and woman makes no differences statistically.

**Figure 2:**
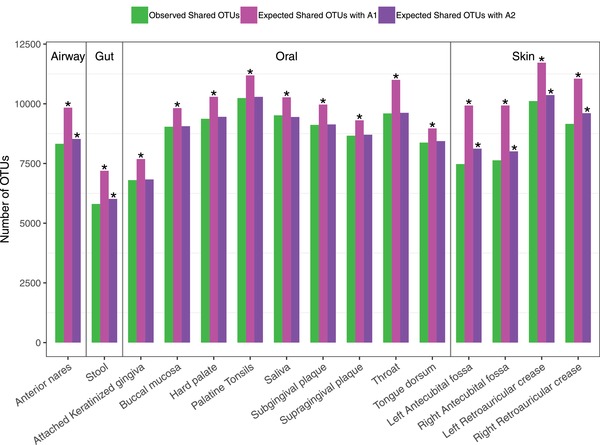
The shared species between the male and female at each of the 15 microbiome sites: Bar in green represents observed shared OTUs (species), bar in magenta represents expected shared OTUs with *A1* algorithm, and bar in purple represents expected shared OTUs with *A2* algorithm. Asterisks indicate that the number of observed shared OTUs between the male and female was significantly smaller than expected by chance (*p* ≤ 0.05). See Table S2–1 (Supporting Information) for the detailed numeric information on the diversity comparisons.

**Figure** **2** and Table S2–1 (Supporting Information) illustrate the results of shared species analysis between the male and female at each of the 15 microbiome sites. With *A1* algorithm (reshuffling reads), the observed number of shared species between the male and female was significantly smaller than that expected by chance in all 15 microbiome sites. With *A2* algorithm (reshuffling samples), the observed number of shared species between the male and female was significantly smaller than that expected by chance in airway, gut, and skin, but the oral sites were exceptions. That is, except for the oral microbiome, there are significant numbers of sex‐specific species in the airway, gut, and skin microbiomes. Since *A2* is more conservative, we take the conclusion from *A2*. These results, in comparison with the previous results of diversity analysis, are particularly interesting. That is, while the community diversities of gut microbiomes exhibited no sex significant difference, their species compositions can be significantly different between sexes!

We also conducted shared species analysis for the five major phyla, similar to the previous diversity analysis (second layer). With phylum *Actinobacteria*, the pattern is similar to the pattern revealed by the previous shared species analysis at the whole community level, except that shared species in the gut (stool) microbiome between the male and female is not influenced by sex. With phyla *Bacteroidetes* and *Firmicutes*, gut microbiome is the only site that showed significant sex difference in terms of the shared species (Table S2–2, Supporting Information).

Table S2–3 (Supporting Information) lists the actually observed shared species between the male and female, as well as the sex‐specific species at each of the 15 microbiome sites.

### 2.3 Sex Difference in the Intersubject Heterogeneity Based on the Extended Power Law

**Figure 3:**
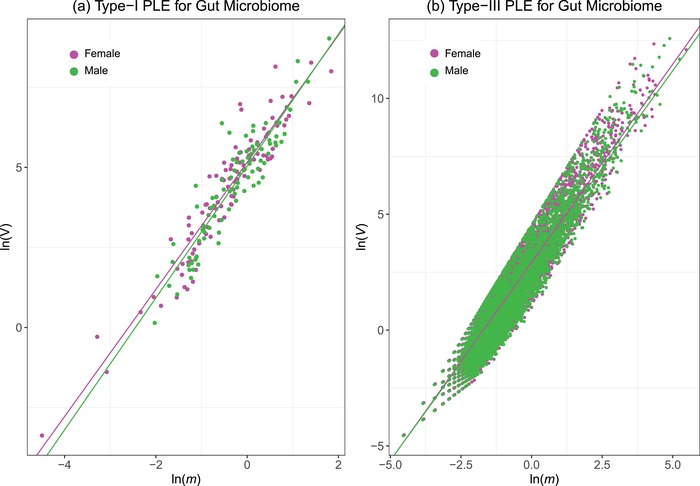
Graphs of fitting Type‐I PLE and Type‐III PLE with the gut microbial samples of the male and female: a) Type‐I PLE; b) Type‐III PLE. The pink points and line represent for the female and the green represent for the male. There were no significant differences in the PLE parameters for the gut microbiome between both the sexes. Similar to the gut microbiome, no PLE scaling parameters were detected in absolute majority of the sites, except for (i) the comparison of buccal mucosa in Type‐I PLE parameters, and (ii) the comparisons of left and right antecubital fossa and right retroauricular crease in Type‐III PLE parameters.

Table S3–1 (Supporting Information) lists the parameters of Type‐I and Type‐III power law extension (PLE) models for the male and female, respectively, at each of the 15 microbiome sites. Table S3–2 (Supporting Information) lists the *p*‐values of the permutation tests for the sex differences in the PLE parameters. **Figure** **3** displays the graphs of Type‐I and Type‐III PLE models, using gut microbiome to illustrate possible sex differences. In nearly all the 15 sites, the PLE scaling parameter (*b*) exhibited no significant sex differences, indicating that the intersubject heterogeneity of the human microbiome is not sex‐specific.

### 2.4 Sex Differences in Diversity‐Scaling Profiles with DAR

**Figure 4:**
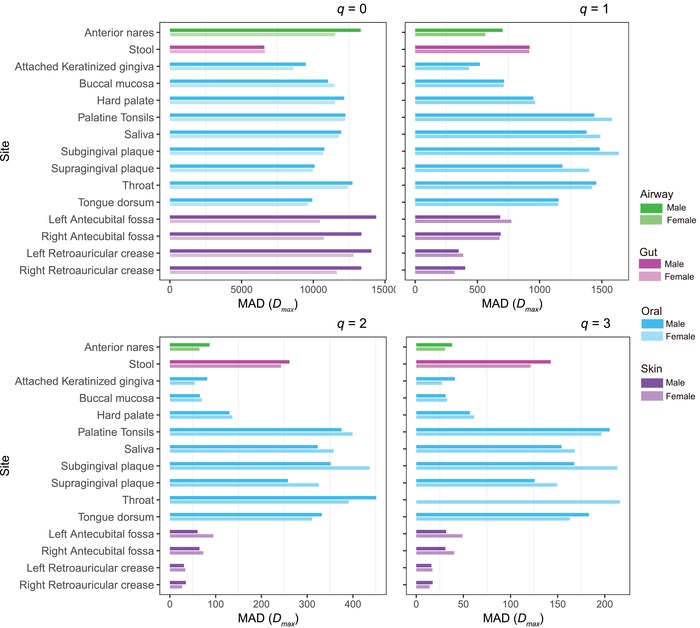
The MAD profiles of the male and female microbiomes at each of the 15‐microbiome sites: Transparency degree of bar indicates gender, the low‐transparency (dark) bar represents for the male, and high‐transparency (light) bar represents for the female. Four different colors indicate four different microbiome locations: red for airway, blue for gut, green for oral, and purple for skin. See Table S4–1 (Supporting Information) for the detailed numeric information on the diversity comparisons.

Table S4–1 (Supporting Information) lists the parameters of the DAR models fitted for each of the 15 microbiome sites of each sex. Table S4–2 (Supporting Information) lists the *p*‐values from the permutation tests for the differences in the DAR parameters between both sexes. No significant sex differences in all DAR parameters were detected. **Figure** **4** displays the maximal accrual diversity (MAD) profile of the male and female microbiomes at each of the 15 microbiome sites, as an example of DAR analysis. Therefore, we conclude that the diversity scaling (change of diversity across a cohort or population) and potential diversity of the human microbiome is not sex‐specific.

### 2.5 Comparing the Properties and Motifs of Basic Species Co‐Occurrence Networks

**Figure 5:**
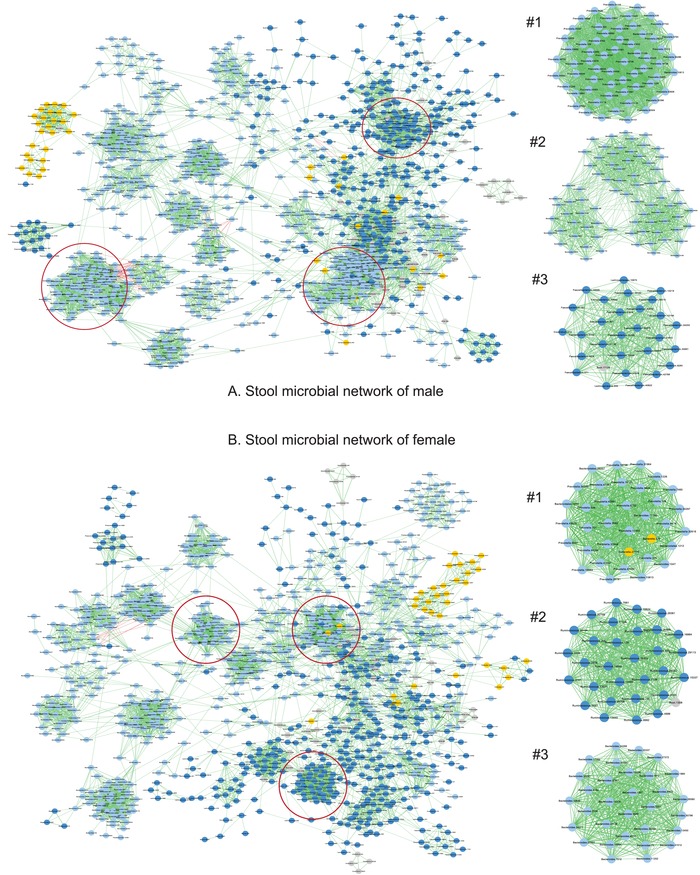
The basic networks of the male and female gut microbiomes: A) The network for the male, B) the network for the female: nodes in light blue—*Bacteroidetes*, nodes in dark blue—*Firmicutes*, nodes in yellow—*Proteobacteria;* edges in red—negative correlations; edges in green—positive correlations.

**Figure** **5** displays the basic SCN of gut microbiome of the male and female, respectively. The top three strongest clusters, from MCODE analysis, were included in the networks. The permutation tests (with 1000 times of resampling) for the sex differences in basic SCN networks revealed mixed results. Overall, in the majority of the sites, basic network properties and motifs did not exhibit significant differences between both sexes (Tables S5–4 and S5–5, Supporting Information).

The P/N ratio, the ratio of positive‐to‐negative links,^26^ exhibited no significant sex differences (Table S5–1, Figure S7, Supporting Information). Some special trios connected with most abundant OTU (MAO) are sex‐specific, and some are not (Tables S5–2 and S5‐3, Supporting Information). In 7 of the 15 sites, sex exerted a significant effect on the occurrences of some special trios. For example, in the case of gut microbiome, the male microbial network had 1491 DLM (double‐link MAO trio), which is 18 times of the number in the female microbial network. These differences confirm that the species interactions or co‐occurrence relationships can be sex‐specific. Table S5–6 (Supporting Information) lists the results of MCODE analysis, which identity strongly connected clusters in a network. These comparisons produce limited insights in our opinion. The CPN and HSN analyses discussed next offer better alternatives to compare both sexes from network perspective.

### 2.6 Shared CPN Analysis between the Male and Female

**Figure 6:**
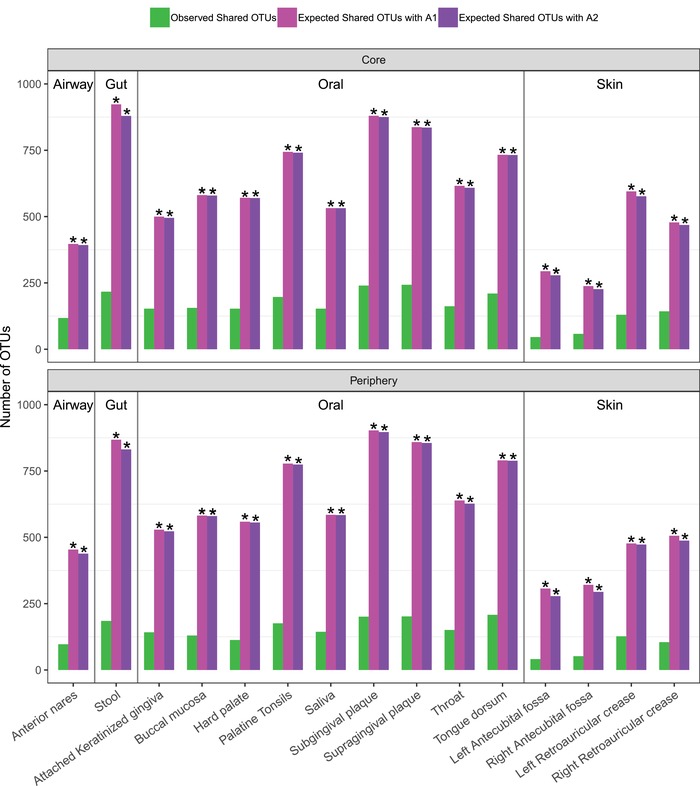
The shared core/periphery network (CPN) analysis between the male and female at each of the 15 microbiome sites: Bar in green represents for the observed shared species, bar in pink represents for the expected shared species with *A1* algorithm, and bar in purple represents for the expected shared species with *A2* algorithm. Asterisks indicate that the number of observed shared species between the male and female was significantly smaller than that expected by chance (*p* ≤ 0.05). See Table S6–1 (Supporting Information) for the detailed numeric information on the diversity comparisons.

**Figure** **6** exhibits the results of the shared core species analysis and shared periphery species analysis, respectively, between the male and female, for each of the 15 microbiome sites, based on the observe‐network strategy. As shown in Table S6–1 (Supporting Information), with both *A1* algorithm (reshuffling reads) and *A2* algorithm (reshuffling samples), the observed numbers of shared core and periphery species were significantly lower than those expected by chance at all 15 microbiome sites. Both the algorithms cross‐verified that, the male and female, each has sex‐specific core or periphery species. Furthermore, the test with the permutated network strategy (Table S6–2, Supporting Information) produced the same conclusions as the observed‐network strategy. That is, the male and female has their sex‐specific, respective core/periphery species, as demonstrated by the reduction of the observed shared core/periphery nodes between both sexes. From Vellend–Hanson synthesis,^29^, ^30^ the shared CPN analysis reveals significantly different selection effects between both sexes from the node perspective. That is, sex can have significant selection effect on microbial species (node) fitness—leading to the different composition of core/periphery species between both sexes.

While the shared core/periphery species are significantly affected by sex at all 15 microbiome sites as explained earlier, the properties of CPN are influenced significantly by sex in 40–70% sites only, depending on specific site and/or specific CPN property (Table S6–3, Supporting Information). This should be expected since the selection effects of sexes may not be sufficiently strong to influence all CPN properties.

We also identified shared core species between both sexes as well as sex‐specific core species (Table S6–4, Supporting Information). Similarly, Table S6–5 (Supporting Information) lists the shared periphery species between both sexes as well as sex‐specific periphery species.

### 2.7 Shared HSN Analysis between the Male and Female

**Figure 7:**
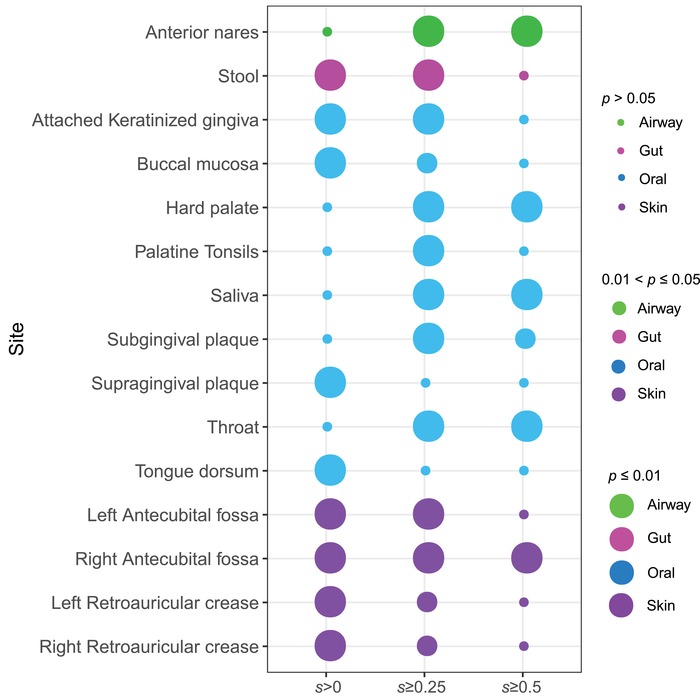
The *p*‐values from the permutation tests for the shared skeletons between the male and female: The color of solid circle indicates microbiome site (i.e., green = airway, magenta = gut, blue = oral, purple = skin). The size of circle represents for the level of *p*‐value: the greater the difference in salience between the male and female, the smaller the *p*‐value, and accordingly, the larger the circle size is. See Table S7–1 (Supporting Information) for the detailed numeric information on shared skeleton analysis.

The shared skeleton analysis can only be performed with the permutated‐network strategy (with 1000 pairs of permutated networks). **Figure** **7** displays the results of the shared skeletons analysis between the male and female for each of the 15 microbiome sites. As shown in Table S7–1 (Supporting Information), when salience value (*s*) *s* ≥ 0.25, except for both sites of tongue dorsum and supragingival plaque, the numbers of shared skeletons, that is, in 13 out of 15 sites, were less than those expected by chance. The observed numbers of shared skeletons with *s* ≥ 0.5 were significantly smaller than those expected by chance in only 6 out of 15 sites, including anterior nares, hard palate, saliva, throat, subgingival plaque, and right antecubital fosa sites. The narrower differences (fewer sites exhibiting differences), when *s* is raised, indicate that both sexes are more homogenous for the more frequently used backbones, which should be expected. That is, the critical or frequently used backbone of gut microbiome (e.g., *s* ≥ 0.5) seems more stable (little difference) between both sexes.

Similar to the CPN analysis, we also tested the differences in the HSN properties between both sexes. Except for one comparison of the skewness, all HSN properties exhibited significant differences between both sexes (Table S7–2, Supporting Information). This also indicates that the influence of sex on the species interactions (network links characterized by HSN) is more prevalent than on the species per se (network nodes characterized by CPN).

The above results indicate that the selection effects of sex on the composition of shared skeletons between both sexes, as well as on the HSN properties are prevalent, similar to the results of the CPN analysis in previous section. That is, the selection effects of sexes can lead to differences in the backbone of species interactions.

According to Li and Ma^31^ testing of the neutral theory with this HMP dataset, the neutrality rate was less than 1% and is not sex‐specific, which suggests that drift, speciation, and dispersal (i.e., three out of the four processes in Vellend–Hanson synthesis) are not sex‐specific. This indicates that host selection (including sex selection) plays a dominant role in driving the community diversity patterns and dynamics, and further verifies that the ecological differences of microgenderome are primarily caused by the selection effects of sex.

## 3 Conclusions

In summary, previous analyses with medical ecology and network science approaches reveal a series of microgenderome characteristics, which can be summarized as seven aspects (**Table** **1**). These characteristics are likely to exert far‐reaching and significant influences on disease susceptibility, and may be responsible for the observed sex differences in some microbiome‐associated diseases, particularly in many autoimmunity diseases. We further postulate that the revealed microgenderome characteristics are primarily caused by the sex difference in selection effects, which are, in turn, shaped/modulated by host physiology (immunity, hormones, gut–brain communications, etc.). Interestingly, these aspects of host physiology in terms of traditional biomedicine are deeply interwoven with emerging medical ecology of human microbiomes because microbiome hosts are essentially the environments of microbiome ecosystems. Indeed, our microgenderome analysis heavily relied on some of the latest advances in medical ecology, bioinformatics, and network science.^15^, ^16^, ^17^, ^20^ The findings and insights from this study present a medical ecology baseline of microgenderome, and provide a references and guideline for mechanistic investigation of sex‐specific susceptibility to certain microbiome‐associated diseases, which, in turn, can be valuable for optimizing their prevention and treatment strategies.

**Table 1:** The ecological and network differences of microgenderome and their implications

Ecological and/or network properties	General assessment on the sex difference	Implications
Species diversity	(i) 7 out of 15 sites exhibited significant sex differences (*M > F* in five sites, and *F > M* in two sites), but some differences may only be detected at phylum or core/periphery level. (ii) At the whole community level, skin microbiome exhibited the most prevalent sex differences (*M > F*), but gut did not surprisingly.	Sex factor should not be ignored in diversity analysis, particular for key phyla such as: *Actinobacteria, Bacteroidetes*, and *Firmicutes*.
Shared species	(i) Except for the oral, there are sex‐specific species in the airway, gut, and skin microbiomes. (ii) With phyla *Bacteroidetes* and *Firmicutes*, gut microbiome is the only site with significant difference in shared species.	Species composition is highly sex‐specific, and there are sex‐specific species for each sex. Our study presented the list of sex‐specific species.
Heterogeneity scaling	Intersubject community heterogeneity scaling (change) is not sex‐specific.	This means sex makes no differences in intersubject community heterogeneity, and diversity changes across cohorts or populations.
Diversity scaling	Diversity scaling and potential diversity are not sex‐specific.	
Basic species co‐occurrence networks (SCN)	(i) “Yin and Yang” are balanced, given that the P/N ratio is not sex‐specific; (ii) There are sex‐specific trio motifs; (iii) Most other basic network properties exhibited mixed results.	The functionalities of those sex‐specific special trios motifs are worthy of further investigations.
Core/periphery network (CPN)	(i) Both observed‐network and permutated network test strategies cross‐verified that core/periphery structures are sex‐specific at all sites. (ii) In 40–70% sites, the CPN properties are influenced significantly by sex, depending on specific site and/or specific CPN property.	CPN and HSN analyses reveal sex‐specific, differential effects among microbial species, which are the selection effects according to Vellend–Hanson synthesis. This is because selection is about the inequality (asymmetry), and CPN/HSN can effectively detect the inequalities from either node or link perspective. Hence, the microgenderome is primarily due to sex‐specific selection effects between man and woman.
High‐salience skeleton network (HSN)	(i) The shared high‐salience skeletons (backbone or critical paths in species interactions) are sex‐specific in all but two oral sites. But more frequently used backbones are less sex‐specific and show much sexual congruity. (ii) Virtually all HSN properties were sex‐specific, exhibited the prevalent sex differences in species interactions.	

## 4 Experimental Section

*HMP Datasets*: The HMP datasets included a cohort of 242 healthy adults, with 129 male and 113 female, each of whom was sampled at 15 (male) and 18 (female) body sites, respectively. The three vaginal sites were excluded for obvious reason. The operational taxonomic unit (OTU) tables (computed from 16S‐rRNA sequencing reads of V1‐V3 region, at 97% similarity or species‐level similarity) as well as the metadata information on the 242 individuals are publicly available at https://www.hmpdacc.org/.^18^

*Approaches, Algorithms, and Computational Procedures*: The medical ecology of the human microbiome can be considered as an interdisciplinary field of medical microbiology, clinical medicine, and theoretical ecology, supported by metagenomics technology, bioinformatics, complexity sciences with objectives to provide the theory and technology for supporting mechanistic/etiological investigations and personalized precision diagnoses and treatments of human microbiome associated diseases.^16^, ^17^ From the state‐of‐the‐art advances in medical ecology, we choose seven approaches (models, algorithms, and procedures) to analyze the microgenderome by reanalyzing the HMP data introduced previously.

*Sex Differences in Microbiome Diversity with Hill Numbers*: The microbiome diversity is quantified using Hill numbers^19^ as follows

(1) $${}^{q}D\;=\;{\left(\sum_{i=1}^{S}p_{i}^{q}\right)}^{1/\left(1-q\right)}$$

where *D* is the diversity, *q* is the order number of diversity, *S* is the number of species, and *p_i_* is the relative abundance of species *i*.^19^ Hill numbers are considered as the most appropriate metrics for alpha‐diversity, because (i) they are in the units of species or species equivalents such as OTUs; (ii) commonly used diversity metrics such as species richness, Shannon entropy, Simpson's index are special cases or functions of Hill numbers; (iii) the rarefaction estimation can be used to deal with the sampling effects. Two tests, nonparametric Wilcoxon test and Cohen's *d*‐statistic for effect sizes, are used to determine the sex differences in diversities to increase the robustness of the tests.^32^

*Shared Species Analysis between the Male and Female*: The number of shared species between the male and female is tested against random effects based on the study of Ma et al.^20^ If there are distinctive species associated with the male or female, then there should be relatively fewer shared species between both the sexes. Alternatively, if the same microbiome is associated with male and female individuals, the distinctive species in each sex would represent random sampling effects, which are especially strong for rare or undersampled taxa, and the number of shared species would be no different than expected by chance (*H*_0_). The shared species analysis compares the composition, or beta diversity^33^ of both sexes, whereas the previous diversity analysis with Hill numbers compares the alpha diversity. Two algorithms (*A1* and *A2*) were used to estimate the number of shared species under *H*_0_. Both *A1* and *A2* were designed based on the principle of the permutation (randomization) test. While *A1* reshuffle the reads, *A2* reshuffle the samples and is more conservative (reliable) in testing the random effects.^20^

*Sex Difference in the Intersubject Heterogeneity Based on the Extended Power Law*: Ma extended Taylor's power law, a classic model for characterizing population spatial aggregation (heterogeneity) and verified by numerous field studies, to the community level by introducing four PLEs.^21^, ^34^, ^35^ Type‐I PLE was proposed to quantify the community spatial (interindividual) heterogeneity, and it has the same mathematical formula with the original Taylor's power law, but with different ecological interpretations, that is

(2) $$V_{s}\;=\;am_{s}^{b}\;\;\;\;\;\;s\;=\;1,2,\ldots ,S$$

where *m*_s_ is the mean of population abundances of all species at the *s*th sampling site (community) (i.e., the mean population size (abundance) per species), *V*_s_ is the corresponding variance, *S* is the number of total sampling sites, *b* is the type‐I PLE parameter for measuring the community spatial (interindividual) heterogeneity, and *a* is a sampling‐related parameter. Whether or not *b* is sex‐specific will be tested with the permutation test based on 1000 times of resampling (see OSI for the algorithm). Similarly, Type‐III PLE was proposed to assess the spatial heterogeneity of the mixed‐species population.^21^

*Sex Differences in Diversity‐Scaling Profiles with DAR*: Since all Hill numbers (Equation (1)) are in the units of species or species equivalents, Ma extended the classic SAR to general DAR, in which diversity is measured with Hill numbers.^22^ The DAR model can use the classic power law function, that is

(3) $${}^{q}D\;=\;cA^{z}$$

where *^q^D* is diversity measured in Hill numbers of *q*th order, *A* is *area*, and *c* and *z* are parameters. A slightly modified PL model, the power law with exponential cutoff (PLEC) model, can also be utilized for DAR modeling, that is

(4) $${}^{q}D\;=\;cA^{z}\exp\left(dA\right)$$

where *d* is a third parameter and should be negative in DAR scaling models, and exp(*dA*) is the exponential decay term that eventually overwhelms the power law behavior at very large value of *A*. Ma derived the MAD based on the PLEC model as

(5) $${}^{q}D_{\max}\;=\;c{\left(-\frac{z}{d}\right)}^{z}\exp\left(-z\right)\;=\;cA_{\max}^{z}\exp\left(-z\right)$$

where *c*, *z*, and *d* are the parameters of the PLEC model for DAR, and *A*_max_ = −*z*/*d*.^22^

According to Ma, MAD includes both local diversity and “dark” or “potential” diversity.^23^ Permutation tests with 1000 times of resampling (see OSI for the algorithm) are conducted to detect sex differences in DAR parameters.

*Comparing the Properties and Motifs of SCN*: To reduce the noise effect of spurious OTUs on network constructions, the OTUs whose total reads from all samples (individuals of the same sex) of a particular site is less than 80 were filtered out. Since the average number of samples (individuals) for each sex at each site is around 80, the OTUs removed from the prescreening operation is equivalent to the so‐called *singleton* who has ≈1 read per sample. For each of the 15 sites sampled, a pair of networks was built, one for each sex. Spearman's correlation coefficients computed with the relative abundance of OTUs (species level, i.e., at 97% similarity cutoff) were adjusted with false discovery rata (FDR) control with *p*‐value = 0.001. The FDR‐adjusted correlation coefficients were fed into Cytoscape (V3.6.1) to visualize the networks and into iGraph R‐package to compute the basic network properties from the species co‐occurrence networks.^36^, ^37^

Besides computing basic network properties, P/N ratio was also computed, special trio motifs were detected, and network clusters were mined using the MCODE plug‐in for Cytoscape.^25^, ^26^ The P/N ratio can be considered as a network property that reflects the balance between positive and negative interactions and was found being influenced by MADs.^26^ The trio‐motifs are essentially the simplest motifs in a complex network and the 15 special trio‐motifs are special because they are directly connected to the MAO or most dominant OTU (MDO), or other with special biomedical significances.^25^ The clusters detected with MCODE are similar to ecological guilds in macrobial ecology.

To test the differences between both the sexes in the network properties, P/N ratio, and special trio‐motifs, for each microbiome site, 1000 pairs of permutated networks were built by pooling together the samples from both the male and female. Permutation tests from the 1000 times of resampling (1000 pairs of permutated networks) were performed to determine the possible differences between both sexes in the network properties and/or motifs (see OSI for the test algorithm).

*Shared CPN Analysis between the Male and Female*: Informally, the network core usually denotes a centrally and densely connected set of network nodes, while the network periphery refers to a sparsely connected, usually noncentral set of nodes that are linked to the core.^27^ Robert May's seminal work proposed that network stability may be achieved either by the development of a nested‐like core/periphery structure, or by network modules.^38^ More recent studies established that network cores promote system robustness and evolvability, which can help system to adapt to large fluctuations of the environment, as well as to noise of intrinsic processes.^15^

The difference between the CPN structures and a number of algorithms for detecting network clusters (modules or communities) is that the latter algorithms usually lack the discrimination of network periphery, that is, the analysis of those nodes that do not belong to the core.^15^, ^27^, ^39^, ^40^, ^41^ In addition, a network usually has multiple modules, but usually only one core. This distinction between core/periphery structure and network module is a major reason why the CPN was chosen for this analysis, since obviously, possibly variable numbers of modules and leftover (scattered) nodes will make the comparisons between both the sexes hardly possible. In other words, the CPN overcomes the limitation of the previous clustering detection with MCODE, which suffered from possibly different numbers of clusters or the inability to pair clusters between both sexes.

According to Csermely et al., a perfect or ideal core/periphery network consists of a fully linked core and a periphery that is fully connected to the core, but none of the periphery nodes are connected with each other.^27^ Formally, let *G* = (*V*, *E*) be an undirected, unweighted graph with *n* nodes and *m* edges, and let *A* = (*a_ij_*) is the adjacency matrix of *G*, where *a_ij_* = *1* if node *i* and node *j* are linked and 0 otherwise. Let δ be a vector of length *n* with entries of 1 or 0, if the corresponding node belongs to the core or the periphery, respectively. Additionally, let *P* = (*p_ij_*) be the adjacency matrix of the ideal or perfect core/periphery network of *n* nodes and *m* edges. The detection of core–periphery structure is an optimization problem to find the vector δ such that the objective function (ρ) achieves its maximum. With the vector δ, it is then trivial to classify nodes into either core or periphery

(6) $$\rho \;=\;\sum_{i,j}A_{ij}P_{ij}$$

The CPN structures reflect the heterogeneity or asymmetry of species (OTUs) from node perspective, which are equivalent to microbial species difference in fitness and exhibit the deterministic selection effects, from the node (species) perspective, in terms of Vellend–Hanson synthesis of community ecology and biogeography.^29^, ^30^

The shared CPN analysis was conducted with two schemes: one uses the so‐termed observed‐network strategy, and another is the permutated‐network strategy. With the former, a pair of networks (one for each sex) was built with their respective (observed) samples without pooling together the samples from both sexes. The resultant core/periphery nodes were permutated 1000 times to test their differences between both sexes. With the latter, the samples from both sexes were first pooled together, and 1000 pairs of random permutations from the combined samples were generated to construct 1000 pairs of permutated networks. Hence, the permutation (randomization) test was indirectly performed with the observed network strategy, and was directly conducted with the permutated‐network strategy. The latter strategy should be more robust but more computation‐intensive than the former. See OSI for the algorithms of both strategies.

*Shared HSN Analysis between the Male and Female*: While core/periphery network distinguishes the different structural and functional roles between core and periphery nodes (species), the HSN makes distinctions among the links (edges). The HSN allows to focus on critical paths (interactions) in complex networks. High salience skeletons or backbones of interactions reduce the number of links in the network while preserving the nodes.^15^, ^28^, ^42^ Grady et al. introduced the concept of link salience, which measures the significance of a link and is based on an ensemble of node‐specific perspectives of the network, and quantifies the extent to which a consensus among nodes exists regarding the importance of a link.^28^ The so‐termed high‐salience skeletons then constitute the backbones (“highways”) of the network. Therefore, the high salience skeletons (links) reflect the heterogeneity or asymmetry of species (OTUs) interactions, or selection effects from the link perspective, in terms of Vellend–Hanson synthesis.^29^, ^30^

Link salience (*s*) is defined based on the notion of shortest paths in weighted networks, for example, the species co‐occurrence network with correlation coefficients as weights. Assume a weighted network defined by weight matrix *w_ij_* and a shortest path between node *x* and *y*, the indicator function can be defined as: σ_*ij*_(*y*,*x*) = 1 if edge (*i*, *j*) is on the shortest path from *x* to *y*, σ_*ij*_(*y*,*x*) = 0, otherwise. A shortest path tree *T*(*x*) rooted at node *x* is described by a matrix with elements: *T_ij_*(*x*) = 1, if $\sum_{y}\sigma_{ij}(y,x)>0$, *T_ij_*(*x*) = 0 otherwise. Link salience *s_ij_* of edge (*i, j*) is computed with the following formula

(7) $$s_{ij}\;=\;\frac{1}{N}\sum_{x}T_{ij}\left(x\right)\;=\;{\left\langle T_{ij}\left(x\right)\right\rangle }_{V}$$

where 〈•〉_*V*_ is the average across the set of root nodes *x*. Since the inverse of correlation coefficient is used as the weight, the shortest path is equivalent to the strongest path in terms of the species interaction (correlation) in this study.

By applying the algorithms for detecting the core/periphery nodes (Equation (6)) and the high‐salience skeletons (Equation (7)) described earlier to the SCNs, the corresponding CPNs (HSNs) were obtained (see ref. 15 for implementation details). Similar to the previous standard SCN analysis, for each site, the samples from both the male and female were randomly mixed and 1000 pairs of permutated CPNs (HSNs) were constructed, in order to (i) perform shared core/periphery analysis, or shared skeleton analysis; (ii) test the sex differences in the properties of CPN (HSN) (see the OSI for the test algorithms).

## Conflict of Interest

The authors declare no conflict of interest.

## Supporting information

Supplementary

Supplementary

Supplementary
