# Supplementary material for: How and Why Men and Women Differ in Their Microbiomes: Medical Ecology and Network Analyses of the Microgenderome
Source: Adv Sci (Weinh). 2019 Oct 23;6(23):1902054. doi: 10.1002/advs.201902054 (PMC6891928; doi:10.1002/advs.201902054)
Supplement: Supplementary file 1 — Supplementary [file ADVS-6-1902054-s001.pdf]

Copyright WILEY-VCH Verlag GmbH & Co. KGaA, 69469 Weinheim, Germany, 2019.

Supporting Information

for *Adv. Sci.,* DOI: 10.1002/advs.201902054

How and Why Men and Women Differ in Their Microbiomes: Medical Ecology and Network Analyses of the Microgenderome

Zhanshan (Sam) Ma and Wendy Li*

Online Supplementary Information (OSI) for:

Ma ZS* & Li W (2019) How and Why Men and Women Differ in Their Microbiomes: Medical Ecology and Network Analyses of the Microgenderome. *Advanced Science*.

The OSI includes three files (two PDF files A & B: "Microgenderome-OSI-File-A.pdf" & "Microgenderome-OSI-File-B.pdf"; one Excel file C "Microgenderome-OSI-File-C.xlsx") available from the Wiley Online Library or from the author.

(A) ***This*** PDF file "Microgenderome-OSI-File-A.pdf" contains the following information: Supplements to "Materials and Methods" Section (including the four algorithms for shared species, core/periphery, skeleton analyses and permutation tests); Supplements to "Diversity comparisons between both the sexes" (supported with Figs S1-S7).

(B) Another PDF file "Microgenderome-OSI-File-B.pdf" contains the following OSI tables: Tables S1-1, S2-1, S2-2, S3-2, S4-2, S5-1, S5-2A, S5-2B, S5-3A, S5-3B, S5-4, S5-5, S6-1, S6-2, S6-3, S7-1, S7-2

(C) Excel file "Microgenderome-OSI-File-C.xlsx" contains the following OSI tables: Tables S1-2, S1-3, S2-3, S3-1, S4-1, S5-6, S6-4, S6-5

The four sets of algorithms for permutation tests and shared species, core/periphery, and skeleton analyses

List of Four Sets of Algorithms:

1. The Shared species analysis algorithms (A1 & A2) were used in two sections: (*i*) shared species analysis (in Section 2) and (*ii*) Shared core/periphery nodes (species) analysis with observed-network strategy (in Section 6).

2. The permutation test algorithm for PL/DAR analyses in Sections 3 & 4

3. The permutation test algorithm for the network parameters (properties) of standard species co-occurrence network (SCN), Trios, P/N ratio (Section 5), as well as the parameters (properties) of the core/periphery network (Section 6) and high-salience skeleton network (Section 7).

4. The algorithms for the shared core/periphery/skeleton network analyses with "permutated network strategy" (in Section 6 for shared core/periphery and in section 7 for shared skeleton, respectively)

1. The Shared species analysis algorithms (A1 & A2) were used in two sections: (*i*) shared species analysis (in Section 2) and (*ii*) Shared core/periphery nodes (species) analysis with observed-network strategy (in Section 6).

Here the so-termed "observed network scheme" is used, in which two networks were constructed, one with the actually observed male samples and another with the observed female samples, respectively.

The two algorithms A1 and A2 were originally designed for shared species analysis first reported in Ma et al. (2019). In the present study, they are adapted for shared core/periphery species analysis

Algorithm A1: The shared core and periphery species analysis through "Random Reassignments" of both OTUs and Samples (Reshuffling reads)

Step (1) Compute the column sums of core species (or periphery) for the male and female groups, respectively, *i.e.,* the total number of population abundances for each core species (or periphery) in male and female groups.

Step (2) Pool together the A reads (individuals from the male group) and B reads (individuals from the female group), and obtained $A+B$ reads (individuals) without caring the *identities* (which core, but each core OTU still keep track of its name or label) or *sources* (from the female or male samples). The structure of core OTU table is gone at this step, regardless their row or column numbers in the original core OTU table (or periphery OTU table).

Step (3) Randomly select A reads for the male group, and recount the number of reads (individuals) for each kind of core OTU (or periphery OTU). The leftover reads are assigned to the female group, and similarly, recount the number of reads (individuals) for each kind of core OTU (or periphery OTU) in the female group.

Step (4) Repeat Step (3) for 1000 times, and compute the number of shared core or periphery between the male and female groups for each repetition.

Step (5) Compute pseudo-p value as follows:
Let D be the times when the number of *shared core* (*or periphery*) from 1000 times of random sampling (*i.e*., random re-assignments) does not exceed the number of *shared core* (*or periphery*) *species observed*. A pseudo p-value can be computed as:

$$p=D/1000$$

If p-value<0.05, then we conclude that the difference in shared core (or periphery) species cannot be attributed to random effect alone, and gender is very likely to have a significant effect on the number of shared species.

Algorithm A2: The Shared Core and Periphery Species Analysis through "Random Reassignments" of Samples only (Reshuffling samples).

Step (1) Pool together all microbiome samples from the male and female groups. That is, mix $a+b$ samples.

Step (2) Randomly select a samples from the mixed samples as the male group, and treat the leftover b samples as the female group.

Step (3) Repeat step (2) 1000 times and compute the shared core and periphery species number between the male and female groups for each time of the random sampling.

Step (4) Compute pseudo-p value
Let D be the times when the number of *shared core and periphery species* from 1000 times of random sampling (*i.e.*, random re-assignments) does not exceed the number of *shared species observed*. A pseudo p-value can be computed as

$$p=D/1000$$

If p-value<0.05, then we conclude that the difference in shared core and periphery species cannot be attributed to random effect alone, and hence the decrease of shared species is most likely caused by gender.

2. The permutation test algorithm for PL/DAR analyses in Sections 3 & 4

(1) Fit the power law (PL) model (or DAR model) to the datasets of male and female groups, respectively, and obtain a pair of parameter sets [b, ln(a)] (or parameters of DAR model), one set for the male group and another set for the female group. This step has no difference from how one regularly fits the power law (or DAR model). From this step, one can obtain the *absolute* difference (delta or Δ) between the male and female groups for each parameter, *e.g.*, $\Delta_b=|b_1-b_2|$.

(2) Pool together all samples from the male and female groups, and randomly permutate the orders of pooled samples, *i.e.,* generating the total permutations of all samples from both the sexes. For example, if there are m samples in male group and n samples in female group, then the number of possible total permutation is $(m+n)!$.

(3) Randomly select 1000 orders out of the total permutations [$(m+n)!$]. For each of the selected orders, designate the first m samples as belonging to male group and the remaining n samples as belonging to female group.

(4) Perform the same PL fitting procedure as in step (1) for each of the selected orders, generating 1000 PL models and corresponding 1000 pairs of parameters sets. Compute the absolute differences of respective parameters for each permutation, respectively, *e.g.*, $\Delta_{pb}=|b_{p1}-b_{p2}|$, where the subscript p notes '*permutation*'. A total of 1000 Δ_{pb} is obtained in this step.

(5) Compute the means and standard deviations of the model parameters from the 1000 permutations, as well as the p-value for permutation test. The p-value is defined as the number of permutations with $\Delta_{pb}>\Delta_b$, divided by the total number of permutations (1000). Obviously, the larger the p-value is, the more likely the treatment effect from permutation is in effect. We choose p=0.05 as the threshold for determining the level of significant difference. That is, if $p\leq 0.05$, we judge that the scaling parameter (b) is different between male and female groups.

3. The permutation test algorithm for the network parameters (properties) of standard species co-occurrence network (SCN), Trios, P/N ratio (Section 5), as well as the parameters (properties) of the core/periphery network (Section 6) and high-salience skeleton network (Section 7).

In this test, for each site, 1000 pairs of permutated networks were built to test the network properties by first pooling together all samples from both sexes at the site.

(1) Constructing standard species co-occurrence network (FDR control with p-value<0.001). Computing the core/periphery and skeleton network properties with species correlation networks of the male and female, respectively. From this step, one obtains the *absolute* difference (delta or Δ) between the male and female for each network parameter, *e.g.*, for parameters b, $\Delta_b=|b_m-b_f|$.

(2) Pool together all samples from both the male and female, and randomly permutate the orders of pooled samples, *i.e.,* generating the total permutations of all samples from both the male and female groups. For example, if there are m samples in male group and n samples in female group, then the number of possible total permutations is $(m+n)!$.

(3) Randomly select 1000 orders out of the total permutations $[(m+n)!]$. For each of the selected orders, designate the first m samples as belonging to male group and the remaining n samples as belonging to female group.

(4) Perform the same computing procedure as in step (1) for each of the selected orders in step (3), generating 1000 pairs of species correlation networks and corresponding 1000 pairs of network parameter sets, respectively, *e.g.*, $\Delta_{pb}=|b_{pm}-b_{pf}|$, where the subscript p notes '*permutation*'. A total of 1000 Δ_{pb} is obtained in this step.

(5) Compute the means and standard deviations of the network parameters from the 1000 permutations, as well as the p-value for permutation test. The p-value is defined as the number of permutations with $\Delta_{pb}>\Delta_b$, divided by the total number of permutations (1000). Obviously, the larger the p-value is, the more likely the treatment effect from permutation is in effect. We choose p=0.05 as the threshold for determining the level of significant difference. That is, if $p\leq0.05$, we judge that the network parameter is significantly different between the male and female groups.

4. The algorithms for the shared core/periphery/skeleton network analyses with "permutated network strategy" (in Section 6 for shared core/periphery and in section 7 for shared skeleton, respectively)

Here the so-termed "permutated network (scheme)" is used, in which 1000 pairs of permutated networks were built for each site, by first pooling together all samples from both the male and female samples at the site.

(1) Assuming there are *a* samples from the male, and *b* samples from the female group, compute the pair-wise Spearman's correlation coefficients for the *a* samples in male and the *b* samples in female, respectively. Use FDR control with p=0.001 to filter out insignificant correlations, and obtain the final correlation relationships for building the species correlation networks for the male and female groups, respectively. From the two species correlation networks, detect the core/periphery nodes and skeletons for each network, and compute the shared core/periphery nodes and skeleton edges between the male and female networks, respectively. This step is no difference from regular core/periphery/skeleton network analysis, until computing the shared core/periphery/skeleton.

The shared core/periphery nodes and skeleton edges obtained from step (1) is the actual or *observed* shared core/periphery nodes and skeleton edges.

(2) Pool together all samples from male and female groups and perform random permutation of the combined (a+b) samples. Treat the first *a* samples as the permutated male group and the leftover *b* samples as the permutated female group.

According to the algorithm in Step (1), compute the shared core/periphery nodes and shared skeleton edges for this specific pair of permutated male and female groups.

(3) Repeat Step (2) for 1000 times, and obtain 1000 sets of shared core/periphery/skeleton numbers.

(4) Compute a pseudo-*p* value. Using the number of shared core nodes as example, assume the shared core nodes from Step (1) is N, the numbers of shared core nodes from the 1000 random permutations in Step (2-3) are $N_1, N_{2,\ldots,} N_{i,\ldots} N_{1000}$, the pseudo-*p* value is the proportion of the permutations with $N_i \leq N$ among 1000 times of permutations. That is, assuming n is the number of times satisfying $N_i \leq N$ in 1000 permutations, $p=n/1000$. Obviously, the larger the *p*-value is, the more likely the treatment effect from permutation is in effect. We choose p=0.05 as the threshold for determining the level of significant difference. That is, if $p \leq 0.05$, we judge that the reduction of shared core, periphery and skeleton between male and female is due to gender rather than to random effects.

Supplement to "Diversity comparisons between both the sexes" supported with Figs S1-S7

We performed the comparisons in species diversity between both the sexes with Hill numbers at three layers. First, the comparison was conducted at the whole community level by computing and comparing the Hill numbers with all species in the community sample. Second, the comparison was conducted for five major phyla respectively, including: *Actinobacteria, Bacteroidetes, Firmicutes, Fusobacteria,* and *Proteobacteria*. Third, the comparison was conducted by distinguishing species as *core* species and *periphery* species, which is supported by the core/periphery network (CPN) analysis. In the main manuscript, we presented the results of diversity comparisons at the community level (*i.e.*, the first layer comparison), here we present the results of the second (five major phyla) and third (core/periphery diversity) layers comparisons.

We further dissect the differences between both sexes by focusing on the five major phyla. **Fig S2, Fig S3,** and **Table S1-2** exhibited the comparisons of *species diversity* in terms of each of the five major phyla, at each of the 15 microbiome sites, and we summarize the following findings:

(*i*) For gut (stool) microbiome, although there was no significant difference in the overall species diversity between the male and female, the Hill numbers (at diversity orders q = 0 & 1) of the *Bacteroidetes* phylum were significant different between the male and female (*M*>*F*) [Fig S2(*b*)].

(*ii*) For the *Actinobacteria*, the significant differences in species diversity between the male and female primarily occurred in the comparisons of the skin microbiome sites (*M*>*F*). At diversity order q = 0, there were significant differences in the species diversity of *Actinobacteria* between both sexes in all skin site, and at q = *1*, there were significant differences in the comparisons of *left antecubital fossa* and *retroauricular creases*. Moreover, in comparison of *anterior nares*, Hill number at q = *1* was significantly different between the male and female (*M*>*F*).

For the *Actinobacteria*, the significant differences in species diversity between the male and female primarily occurred in the comparisons of the skin microbiome sites (*M*>*F*) and the pattern is similar with the overall species diversity revealed previously. This general congruity between *Actinobacteria* and the skin microbiome prompt us the critical importance of *Actinobacteria* in determining the sex-difference.

(*iii*) The Hill numbers of the *Firmicutes* were significantly different between male and female in the comparison of saliva (*M*<*F*), and left retroauricular crease (*M*>*F*).

(*iv*) *Fusobacteria* exhibited significant sex-specific difference at *palatine tonsils*. *Proteobacteria* exhibited significant sex-specific difference at *attached keratinized gingiva*, *hard palate*, and *tongue dorsum.*

We also compared the species diversity by distinguish the species as core and periphery species. **Fig S4, Fig S5** and **Fig S6** displayed the comparisons of *core-species* or *periphery-species* diversity between the male and female at each of the 15-microbiome sites, respectively, and the detailed results were listed in **Table S1-3**. We summarize the following findings:

(*i*) *Skin microbiome*: Whether it is core or periphery structure, the male exhibited significant higher diversity than the female at all four skin sites. That is, *M>F* for all skin sites, at core, periphery and the whole community scales.

(*ii*) *Airway microbiome*: In the core structure, the Hill numbers at all four diversity orders (*q*=0-3) exhibited significant sex-specific differences (*M>F*). In the periphery structure, the Hill numbers at diversity *q*=0 &1 exhibited sex-specific differences (*M>F*).

(*iii*) *Gut microbiome*: For the gut (stool) microbiome, the Hill number of core-species was significantly different between the male and female (*M>F*) at order *q* = 1, 2 & 3, and the Hill numbers of periphery-species were significant different between male and female (*M>F*) at diversity orders *q=0*. At diversity order *q*=1, both Wilcox and d-statistic tests generated conflicting evidence. Therefore, while the gut microbiome did not exhibit sex-specific difference at the whole community level, the core and periphery structures displayed sex-specific differences.

(*iv*) *Oral microbiome*: For the core species, there were significant differences in the Hill numbers between the male and female in 4 out of the 9 sites of the oral microbiome (10 comparisons), including *attached keratinized gingival* at *q*=0 & 1, *buccal mucosa* at *q*=1, 2 & 3, *palatine tonsils* at *q*=0, and *tongue dorsum* at *q*=0-3. For the periphery-species, there were significant differences in the Hill numbers between the male and female in 7 out of the 9 sites of the oral microbiome (13 comparisons), including *attached keratinized gingival* at *q*=2 & 3, *buccal mucosa* at *q*=1, hard palate at *q*=0 & 1, *palatine tonsils* at *q*=0, *subgingival plaque* at *q*=0, *supragingival plaque* at *q*=1-3, and *tongue dorsum* at *q*=1-3.

List of Supplementary Figures

Fig S1. **The effect size (*d*=**male–female**) for comparing the community diversity in Hill numbers (at *q*=0-3) of male *vs.* female**: The positive effect size (the bars on the right side) indicates that the male has a higher diversity, and vice versa, the negative *d* (the bars on the right side) indicates a lower male diversity. Asterisks (*) indicate the significant difference (p<0.05) based on Cohen's (1988) *d*-statistic.

Fig S2. **The comparison of species diversity of 5 major phyla in terms of the Hill numbers (at *q*=0~3) of the male *vs.* female**: (a) *Actinobacteria* (b) *Bacteroidetes* (c) *Firmicutes* (d) *Fusobacteria* and (e) *Proteobacteria.* Solid circles with different color represent for different microbiome sites (*i.e.*, green=airway, magenta=gut, blue=oral, purple=skin). Circle size represents for the size of *p*-value from **Wilcoxon test**; the greater the diversity difference, the smaller the *p*-value, and the larger the circle size is accordingly. The farther from the 45° line (equal diversity of the male *vs*. female), and the larger the diversity difference between the male and female. *See* Table S2 for the detailed numeric information on the diversity comparisons.

Fig S3. **The effect size (*d*=**male-female**) for comparing the species diversity of five major phyla in terms of the Hill numbers (at *q*=0-3) of male *vs.* female**: Bar color indicates the diversity order. The positive effect size (the bars on the right side) indicates that the male has a higher diversity, and vice versa, the negative *d* (the bars on the right side) indicates a lower male diversity. Asterisks (*) indicate the significant difference (p<0.05) based on Cohen's (1988) *d*-statistic.

Fig S4. **The comparison of core-species diversity in Hill numbers (at *q*=0~3) of the male *vs.* female**: Solid circles with different color represent for different microbiome sites (*i.e.*, green=airway, magenta=gut, blue=oral, purple=skin). Circle size represents for the size of *p*-value from **Wilcoxon test**; the greater the diversity difference, the smaller the *p*-value, and the larger the circle size is accordingly. The farther from the 45° line (equal diversity of the male *vs*. female), and the larger the diversity difference between the male and female. *See* Table S3 for the detailed information of the diversity comparisons.

Fig S5. **The comparison of periphery-species diversity in Hill numbers (at *q*=0~3) of the male *vs.* female**: Solid circles with different color represent for different microbiome sites (*i.e.*, green=airway, magenta=gut, blue=oral, purple=skin). Circle size represents for the size of *p*-value from **Wilcoxon test**; the greater the diversity difference, the smaller the *p*-value, and the larger the circle size is accordingly. The farther from the 45° line (equal diversity of the male *vs*. female), and the larger the diversity difference between the male and female. *See* Table S3 for the detailed information of the diversity comparisons.

Fig S6. **The effect size (*d*=**male-female**) for comparing core-species diversity and periphery-species diversity, respectively, in Hill numbers (at *q*=0-3) of the male *vs.* female**: The positive effect size (the bars on the right side) indicates that the male has a higher diversity, and vice versa, the negative *d* (the bars on the right side) indicates a lower male diversity. Asterisks (*) indicate the significant difference (p<0.05) based on Cohen's (1988) *d*-statistic.

Fig S7. The P/N ratios in the microbial networks of male and female at each of 15 microbiome sites: the color indicates the gender (pink: female, blue: male).

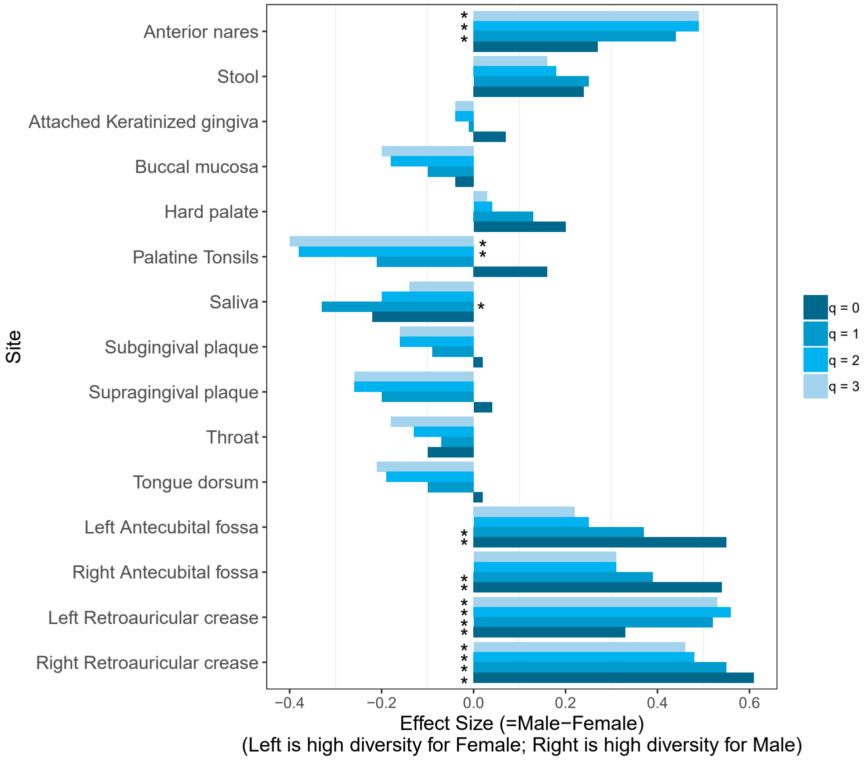

Fig S1. **The effect size (*d*=**male–female**) for comparing the community diversity in Hill numbers (at *q*=0-3) of male *vs*. female**: The positive effect size (the bars on the right side) indicates that the male has a higher diversity, and vice versa, the negative *d* (the bars on the right side) indicates a lower male diversity. Asterisks (*) indicate the significant difference (p<0.05) based on Cohen's (1988) *d*-statistic.

(*a*)

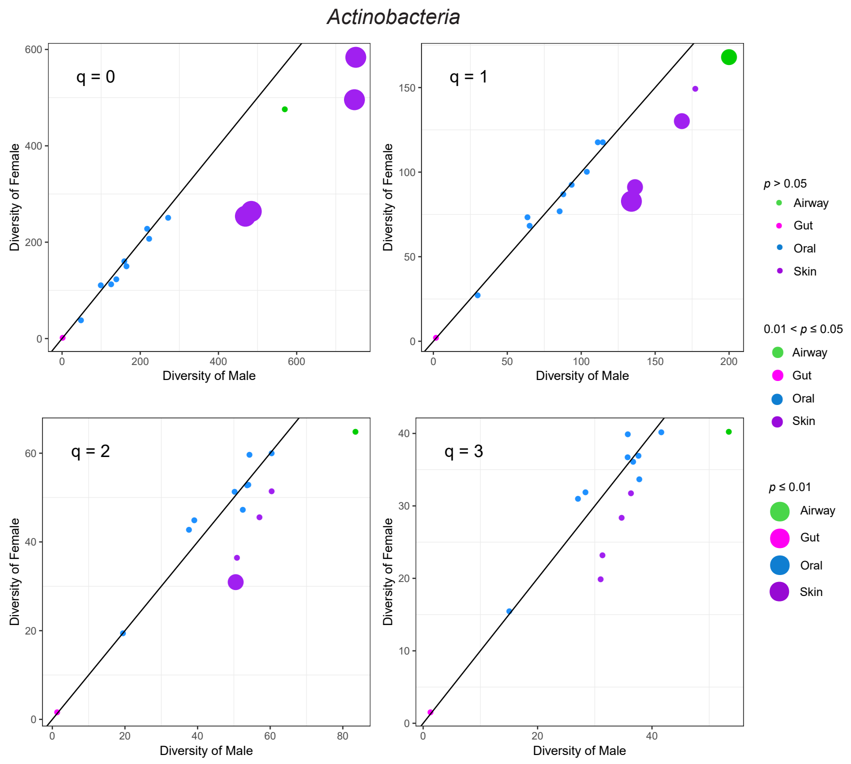

Actinobacteria
q = 0
q = 1
q = 2
q = 3
Diversity of Female
Diversity of Male
p > 0.05
Airway
Gut
Oral
Skin
0.01 < p ≤ 0.05
Airway
Gut
Oral
Skin
p ≤ 0.01
Airway
Gut
Oral
Skin

(*b*)

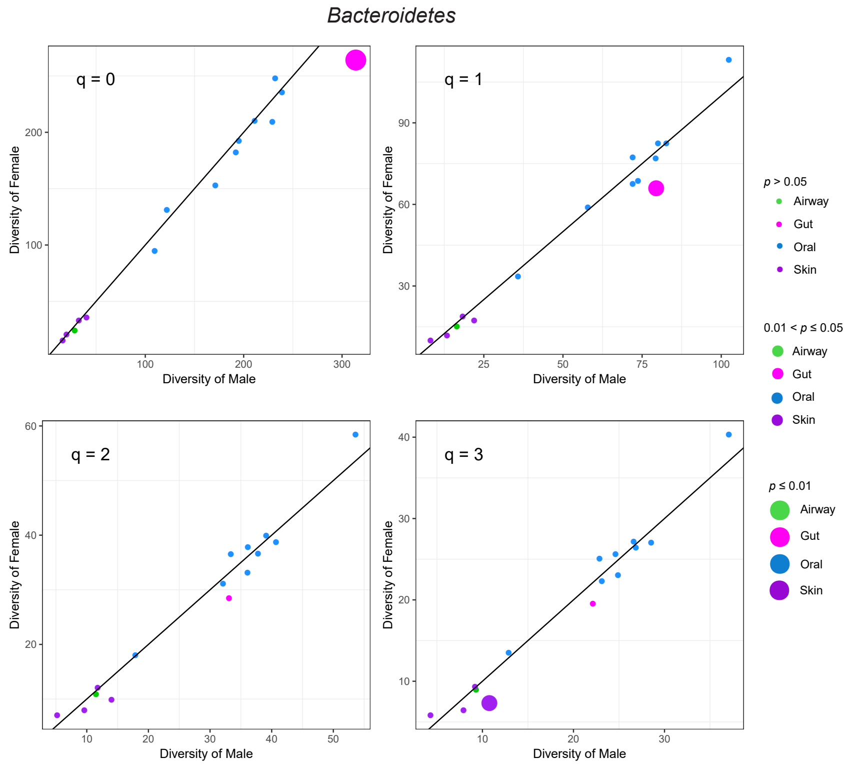

Bacteroidetes
q = 0
q = 1
q = 2
q = 3
Diversity of Female
Diversity of Male
p > 0.05
Airway
Gut
Oral
Skin
0.01 < p ≤ 0.05
Airway
Gut
Oral
Skin
p ≤ 0.01
Airway
Gut
Oral
Skin

(c)

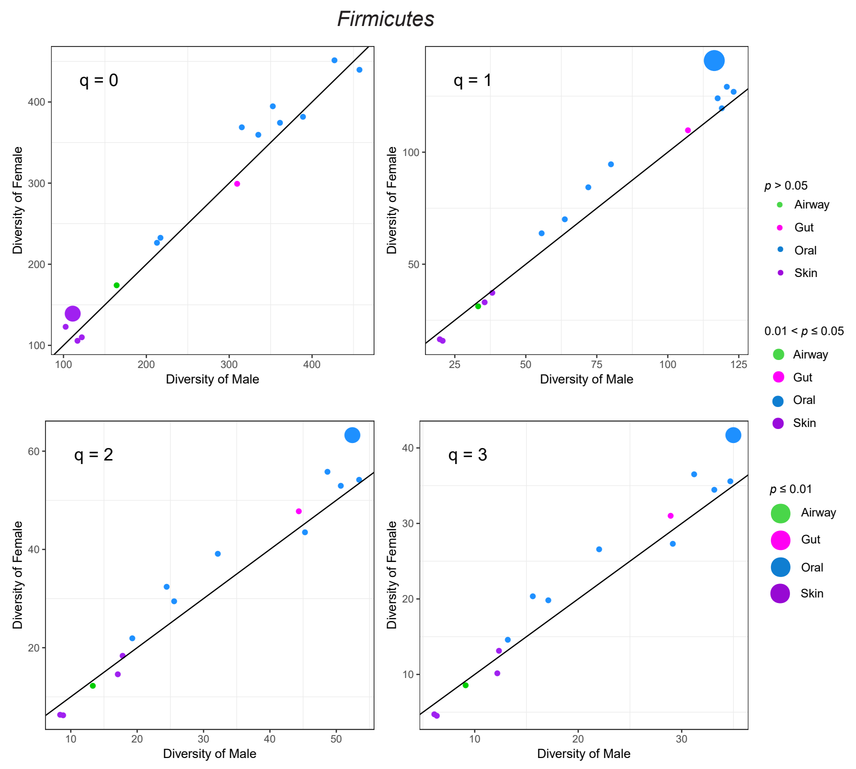
Firmicutes
q = 0
q = 1
q = 2
q = 3
Diversity of Female
Diversity of Male
p > 0.05
Airway
Gut
Oral
Skin
0.01 < p ≤ 0.05
Airway
Gut
Oral
Skin
p ≤ 0.01
Airway
Gut
Oral
Skin

(d)

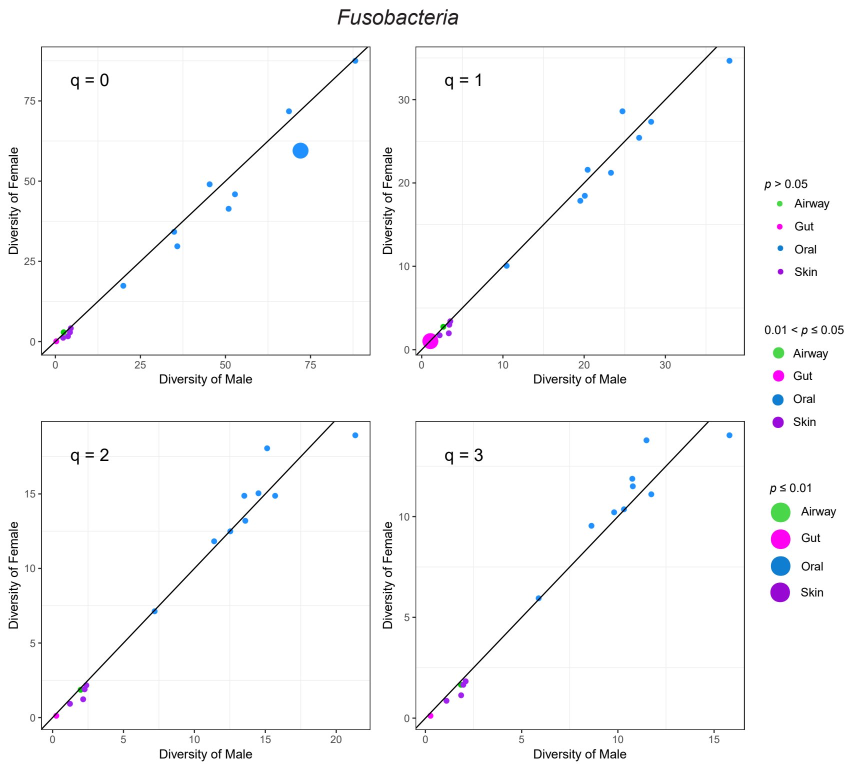
Fusobacteria
q = 0
q = 1
q = 2
q = 3
Diversity of Female
Diversity of Male
p > 0.05
Airway
Gut
Oral
Skin
0.01 < p ≤ 0.05
Airway
Gut
Oral
Skin
p ≤ 0.01
Airway
Gut
Oral
Skin

(*e*)

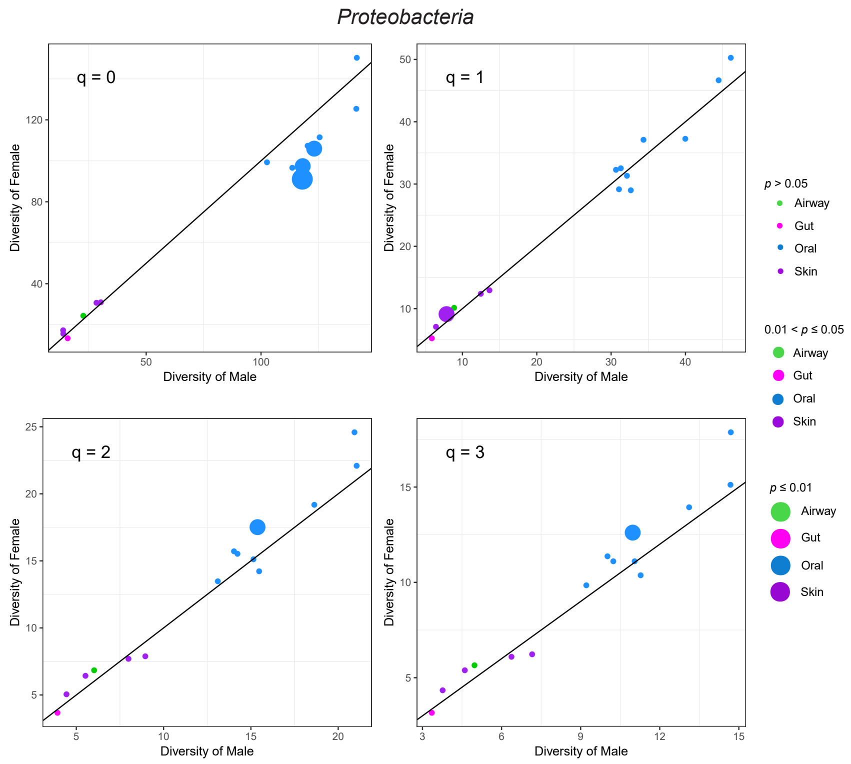

Fig S2. **The comparison of species diversity of 5 major phyla in terms of the Hill numbers (at *q*=0~3) of the male *vs*. female**: (a) *Actinobacteria* (b) *Bacteroidetes* (c) *Firmicutes* (d) *Fusobacteria* and (e) *Proteobacteria.* Solid circles with different color represent for different microbiome sites (*i.e.*, green=airway, magenta=gut, blue=oral, purple=skin). Circle size represents for the size of *p*-value from **Wilcoxon test**; the greater the diversity difference, the smaller the *p*-value, and the larger the circle size is accordingly. The farther from the 45° line (equal diversity of the male *vs*. female), and the larger the diversity difference between the male and female. *See* Table S2 for the detailed numeric information on the diversity comparisons.

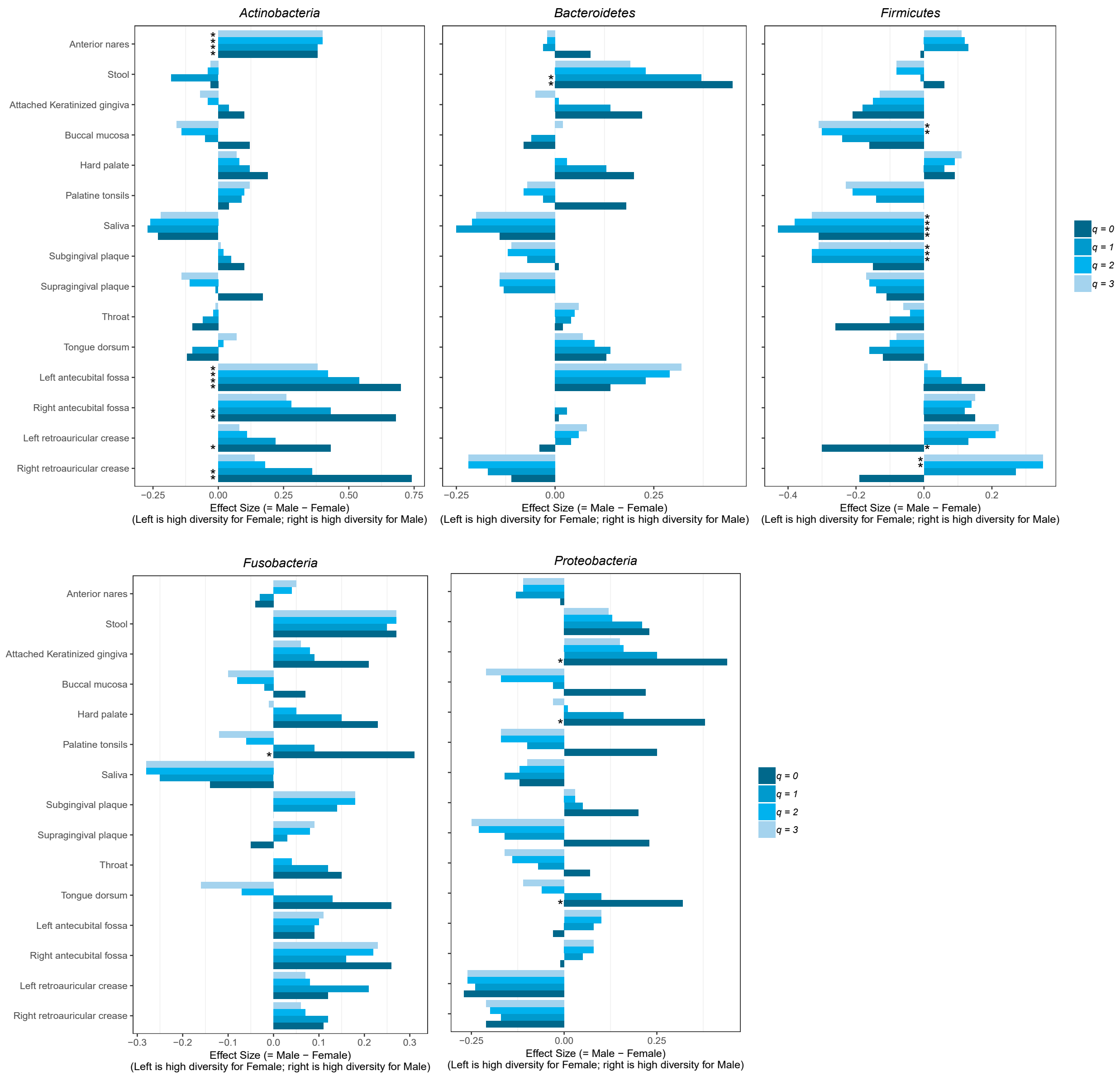

Fig S3. **The effect size (*d*=**male-female**) for comparing the species diversity of five major phyla in terms of the Hill numbers (at *q*=0-3) of male *vs***. **female**: Bar color indicates the diversity order. The positive effect size (the bars on the right side) indicates that the male has a higher diversity, and vice versa, the negative *d* (the bars on the right side) indicates a lower male diversity. Asterisks (*) indicate the significant difference (*p*<0.05) based on Cohen's (1988) *d*-statistic.

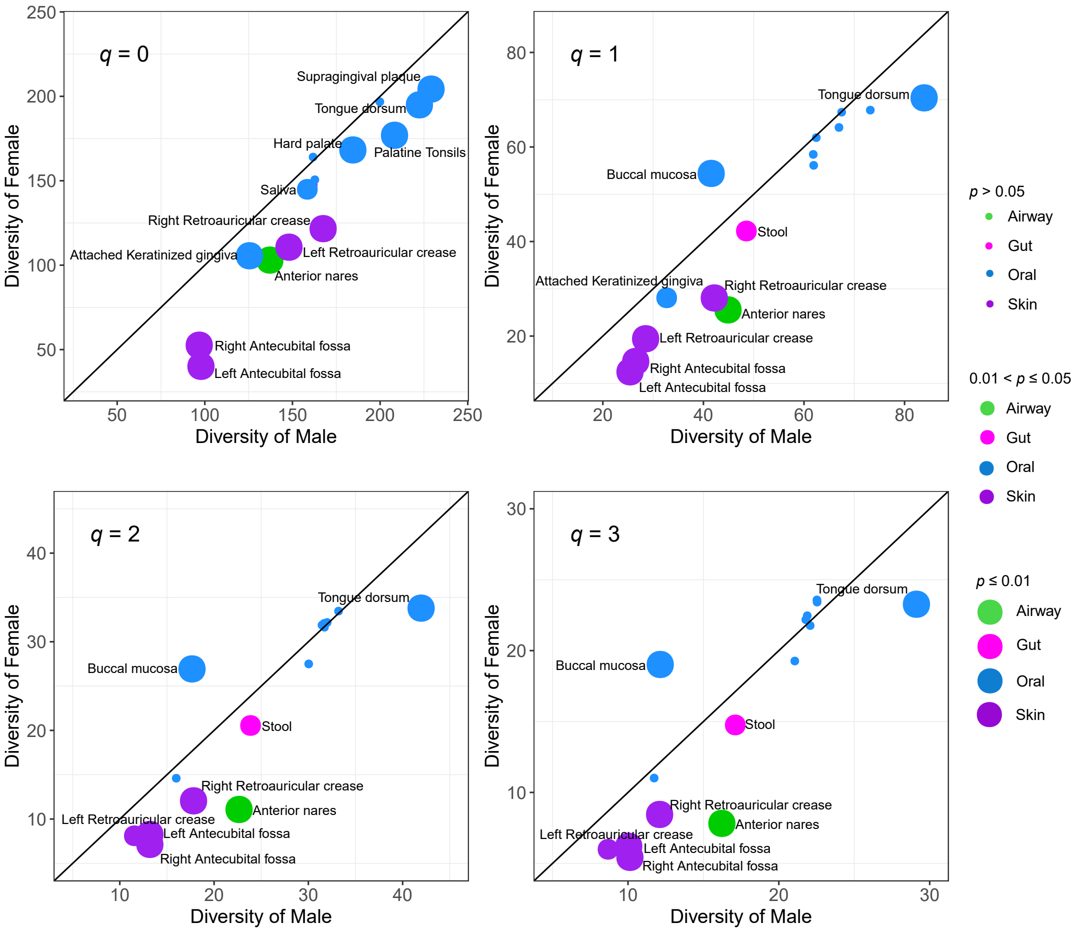

Fig S4. **The comparison of core-species diversity in Hill numbers (at *q*=0~3) of the male *vs.* female**: Solid circles with different color represent for different microbiome sites (*i.e.*, green=airway, magenta=gut, blue=oral, purple=skin). Circle size represents for the size of *p*-value from **Wilcoxon test**; the greater the diversity difference, the smaller the *p*-value, and the larger the circle size is accordingly. The farther from the 45° line (equal diversity of the male *vs.* female), and the larger the diversity difference between the male and female. *See* Table S3 for the detailed information of the diversity comparisons.

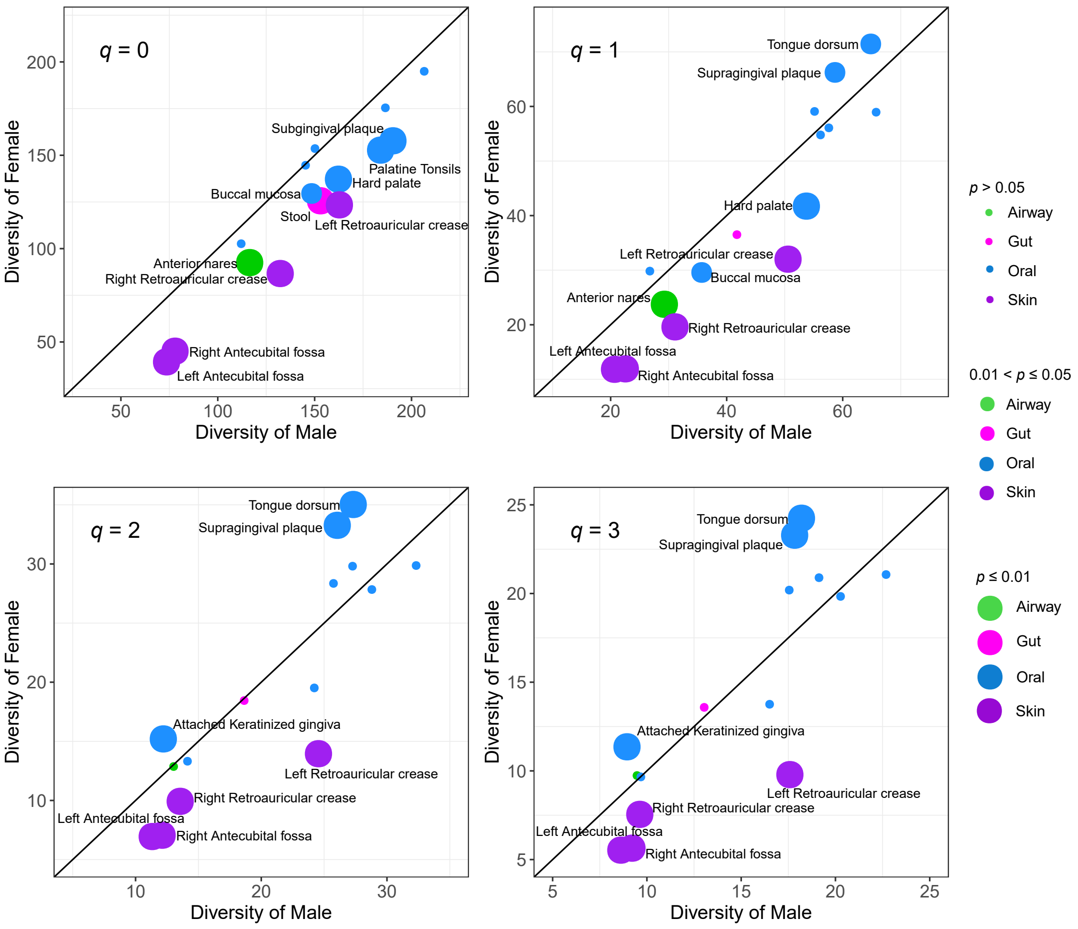

Fig S5. **The comparison of periphery-species diversity in Hill numbers (at *q*=0~3) of the male *vs*. female**: Solid circles with different color represent for different microbiome sites (*i.e*., green=airway, magenta=gut, blue=oral, purple=skin). Circle size represents for the size of *p*-value from **Wilcoxon test**; the greater the diversity difference, the smaller the *p*-value, and the larger the circle size is accordingly. The farther from the 45° line (equal diversity of the male *vs*. female), and the larger the diversity difference between the male and female. *See* Table S3 for the detailed information of the diversity comparisons.

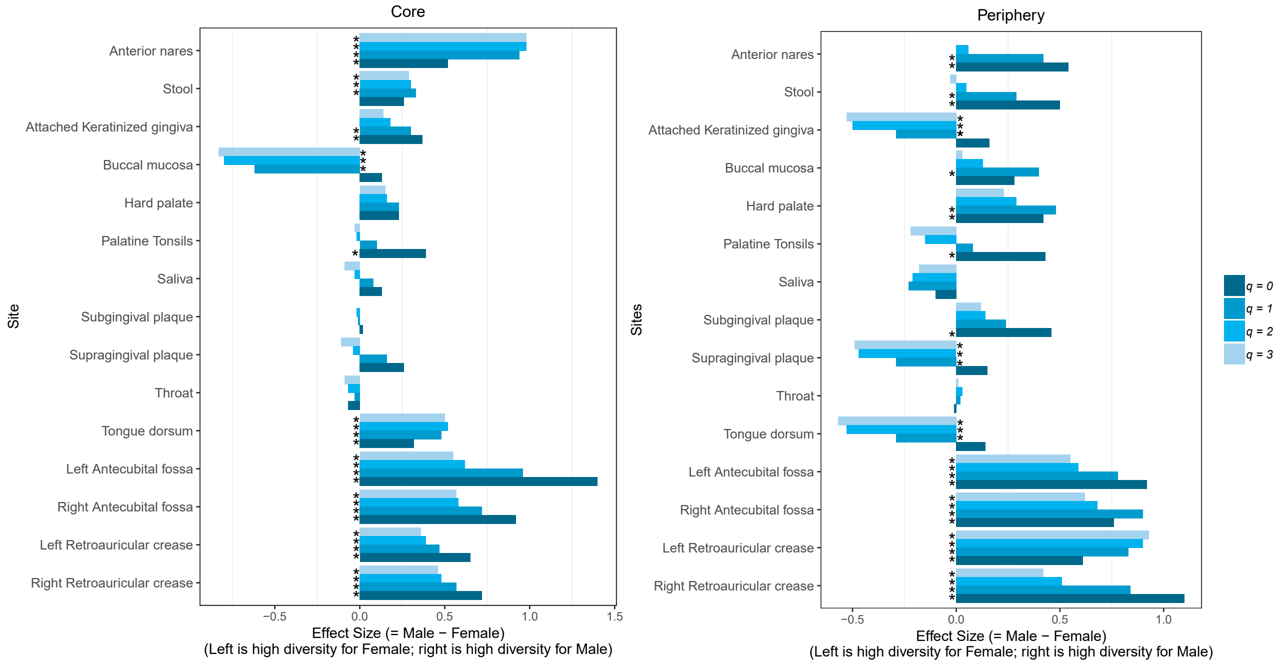

Fig S6. **The effect size (*d*=**male-female**) for comparing core-species diversity and periphery-species diversity, respectively, in Hill numbers (at *q*=0-3) of the male *vs***. **female**: The positive effect size (the bars on the right side) indicates that the male has a higher diversity, and vice versa, the negative *d* (the bars on the right side) indicates a lower male diversity. Asterisks (*) indicate the significant difference (p<0.05) based on Cohen's (1988) *d*-statistic.

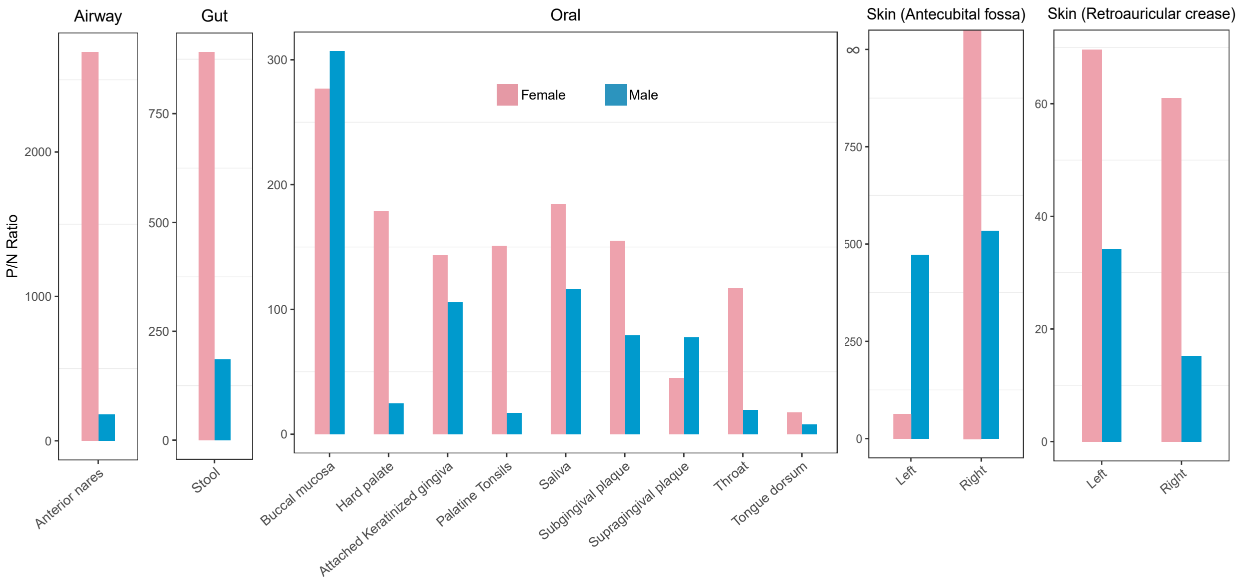

Fig S7. The P/N ratios in the microbial networks of male and female at each of 15 microbiome sites: the color indicates the gender (pink: female, blue: male).
