# Supplementary material for: How and Why Men and Women Differ in Their Microbiomes: Medical Ecology and Network Analyses of the Microgenderome
Source: Adv Sci (Weinh). 2019 Oct 23;6(23):1902054. doi: 10.1002/advs.201902054 (PMC6891928; doi:10.1002/advs.201902054)
Supplement: Supplementary file 2 — Supplementary [file ADVS-6-1902054-s002.pdf]

Copyright WILEY-VCH Verlag GmbH & Co. KGaA, 69469 Weinheim, Germany, 2019.

Supporting Information

for *Adv. Sci.,* DOI: 10.1002/advs.201902054

How and Why Men and Women Differ in Their Microbiomes: Medical Ecology and Network Analyses of the Microgenderome

Zhanshan (Sam) Ma and Wendy Li*

Online Supplementary Information (OSI) for:

Ma ZS* & Li W (2019) How and Why Men and Women Differ in Their Microbiomes: Medical Ecology and Network Analyses of the Microgenderome. *Advanced Science*.

The OSI includes three files (two PDF files A & B: "Microgenderome-OSI-File-A.pdf" & "Microgenderome-OSI-File-B.pdf"; one Excel file C "Microgenderome-OSI-File-C.xlsx") available from the Wiley Online Library or from the author.

(A) Another PDF file "Microgenderome-OSI-File-A.pdf" contains the following information: Supplements to "Materials and Methods" Section (including the four algorithms for shared species, core/periphery, skeleton analyses and permutation tests); Supplements to "Diversity comparisons between both the sexes" (supported with Figs S1-S7).

(B) ***This*** PDF file "Microgenderome-OSI-File-B.pdf" contains the following OSI tables: Tables S1-1, S2-1, S2-2, S3-2, S4-2, S5-1, S5-2A, S5-2B, S5-3A, S5-3B, S5-4, S5-5, S6-1, S6-2, S6-3, S7-1, S7-2

(C) Excel file "Microgenderome-OSI-File-C.xlsx" contains the following OSI tables: Tables S1-2, S1-3, S2-3, S3-1, S4-1, S5-6, S6-4, S6-5

1. Sex differences in microbiome diversity with Hill numbers

Table S1-1. The *mean* and *standard error* of the community diversity (in the Hill numbers) for the male and female at each of the 15 microbiome sites, the results (*d*-statistic and *p*-value) of the effect-size test on diversity, as well as the results of Wilcoxon test (Note the highlighted cells in yellow contain tests with significant difference between both the sexes, with *p*-value < 0.05).

Sample Sites		Treatments		*q*=0	*q*=1	*q*=2	*q*=3
Airway	Anterior nares	Male	Mean	791.247	192.705	63.363	38.113
			Std. Err.	46.420	13.370	7.260	4.816
		Female	Mean	707.056	144.693	39.356	23.084
			Std. Err.	48.957	12.332	4.110	2.347
		Male vs. Female	Cohen's *d*	0.270	0.440	0.490	0.490
			p-value of *d*	0.100	0.010	0.000	0.000
			p-value of Wilcoxon test	0.420	0.015	0.008	0.007
Gut	Stool	Male	Mean	661.337	152.585	53.187	33.328
			Std. Err.	30.316	6.990	2.996	1.988
		Female	Mean	599.033	136.333	48.516	30.742
			Std. Err.	34.277	7.193	3.190	2.128
		Male vs. Female	Cohen's *d*	0.240	0.250	0.180	0.160
			p-value of *d*	0.100	0.090	0.220	0.280
			p-value of Wilcoxon test	0.094	0.106	0.146	0.180
Oral	Attached Keratinized gingiva	Male	Mean	637.404	116.270	37.720	24.073
			Std. Err.	29.942	5.977	2.458	1.544
		Female	Mean	607.310	120.597	40.477	25.633
			Std. Err.	25.272	8.615	3.548	2.066
		Male vs. Female	Cohen's *d*	0.070	-0.010	-0.040	-0.040
			p-value of *d*	0.640	0.930	0.800	0.790
			p-value of Wilcoxon test	0.606	0.760	0.861	0.879
	Buccal mucosa	Male	Mean	848.421	188.261	50.894	29.313
			Std. Err.	37.739	10.274	3.785	2.123
		Female	Mean	855.670	203.332	62.383	36.262
			Std. Err.	43.534	13.332	5.967	3.379
		Male vs. Female	Cohen's *d*	-0.040	-0.100	-0.180	-0.200
			p-value of *d*	0.810	0.510	0.220	0.180
			p-value of Wilcoxon test	0.774	0.807	0.579	0.460
	Hard palate	Male	Mean	972.337	269.741	89.684	52.913
			Std. Err.	49.559	11.832	6.375	4.108
		Female	Mean	891.674	260.545	88.785	52.288
			Std. Err.	40.595	13.549	6.872	4.252
		Male vs. Female	Cohen's *d*	0.200	0.130	0.040	0.030
			p-value of *d*	0.190	0.390	0.770	0.820
			p-value of Wilcoxon test	0.513	0.739	0.812	0.856
	Palatine Tonsils	Male	Mean	984.628	264.770	84.628	48.938
			Std. Err.	45.051	9.764	4.449	2.756
		Female	Mean	906.228	290.104	105.822	62.563
			Std. Err.	34.367	11.384	5.796	3.627
		Male vs. Female	Cohen's *d*	0.160	-0.210	-0.380	-0.400
			p-value of *d*	0.280	0.150	0.010	0.010
			p-value of Wilcoxon test	0.162	0.136	0.009	0.009
	Saliva	Male	Mean	899.506	300.680	114.191	68.591
			Std. Err.	47.240	13.761	6.448	4.186
		Female	Mean	992.920	347.047	128.923	75.974
			Std. Err.	51.722	15.991	8.028	5.504
		Male vs. Female	Cohen's *d*	-0.220	-0.330	-0.200	-0.140
			p-value of *d*	0.160	0.040	0.200	0.380
			p-value of Wilcoxon test	0.176	0.037	0.192	0.428
	Subgingival plaque	Male	Mean	931.821	275.532	97.438	58.112
			Std. Err.	40.856	10.769	4.933	3.205
		Female	Mean	917.034	285.208	105.553	62.994
			Std. Err.	40.154	11.758	5.542	3.396
		Male vs. Female	Cohen's *d*	0.020	-0.090	-0.160	-0.160
			p-value of *d*	0.870	0.530	0.280	0.270
			p-value of Wilcoxon test	0.822	0.760	0.352	0.288
	Supragingival plaque	Male	Mean	935.646	258.314	89.334	53.291
			Std. Err.	42.681	12.132	5.555	3.489
		Female	Mean	918.935	285.018	105.783	64.008
			Std. Err.	40.363	13.318	6.597	4.252
		Male vs. Female	Cohen's *d*	0.040	-0.200	-0.260	-0.260
			p-value of *d*	0.790	0.180	0.080	0.070
			p-value of Wilcoxon test	0.872	0.107	0.050	0.050
	Throat	Male	Mean	898.899	287.803	100.857	58.583

			Std. Err.	42.334	10.880	5.164	3.302
		Female	Mean	927.864	290.503	107.328	64.534
			Std. Err.	39.993	10.531	5.696	3.819
		Male vs. Female	Cohen's *d*	-0.100	-0.070	-0.130	-0.180
			p-value of *d*	0.500	0.660	0.400	0.260
			p-value of Wilcoxon test	0.532	0.842	0.463	0.323
	Tongue dorsum	Male	Mean	981.357	279.823	93.665	54.699
			Std. Err.	46.183	9.113	4.211	2.785
		Female	Mean	967.772	291.934	103.059	61.085
			Std. Err.	40.664	10.498	4.848	3.170
		Male vs. Female	Cohen's *d*	0.020	-0.100	-0.190	-0.210
			p-value of *d*	0.890	0.500	0.190	0.150
			p-value of Wilcoxon test	0.967	0.261	0.167	0.167
Skin	Left Antecubital fossa	Male	Mean	672.728	161.022	52.961	31.247
			Std. Err.	53.874	16.325	7.681	4.519
		Female	Mean	441.219	114.574	37.108	22.518
			Std. Err.	52.941	14.639	4.819	2.725
		Male vs. Female	Cohen's *d*	0.550	0.370	0.250	0.220
			p-value of *d*	0.000	0.030	0.140	0.190
			p-value of Wilcoxon test	0.000	0.020	0.336	0.460
	Right Antecubital fossa	Male	Mean	671.779	164.105	56.637	34.485
			Std. Err.	51.930	17.629	8.428	5.103
		Female	Mean	443.710	116.699	37.888	22.952
			Std. Err.	47.734	14.697	5.235	3.094
		Male vs. Female	Cohen's *d*	0.540	0.390	0.310	0.310
			p-value of *d*	0.000	0.020	0.070	0.070
			p-value of Wilcoxon test	0.001	0.022	0.124	0.135
	Left Retroauricular crease	Male	Mean	902.526	180.701	53.707	31.102
			Std. Err.	59.080	13.789	5.768	3.385
		Female	Mean	765.831	118.538	28.898	17.276
			Std. Err.	52.119	10.366	3.361	1.939
		Male vs. Female	Cohen's *d*	0.330	0.520	0.560	0.530
			p-value of *d*	0.030	0.000	0.000	0.000
			p-value of Wilcoxon test	0.029	0.001	0.001	0.002
	Right Retroauricular crease	Male	Mean	884.714	165.505	47.293	27.507
			Std. Err.	49.250	11.778	4.947	2.883
		Female	Mean	653.933	109.031	28.140	16.839
			Std. Err.	42.083	10.304	3.307	1.922
		Male vs. Female	Cohen's *d*	0.610	0.550	0.480	0.460
			p-value of *d*	0.000	0.000	0.000	0.000
			p-value of Wilcoxon test	0.000	0.000	0.002	0.004

Table S1-2. The *mean* and *standard error* of the species diversity (in the Hill numbers) of 5 main phyla for the male and female at each of the 15 microbiome sites, the results (*d*-statistic and *p*-value) of the effect-size test on diversity, as well as the results of Wilcoxon test (Note the highlighted cells in yellow contain tests with significant difference between both the sexes, with *p*-value < 0.05).
(*This table was listed in Excel File:* Microgenderome-OSI-File-C.xlsx)

Table S1-3. The *mean* and *standard error* of the species diversity (in the Hill numbers) of the core and periphery OTUs for the male and female at each of the 15 microbiome sites, the results (*d*-statistic and *p*-value) of the effect-size test on diversity, as well as the results of Wilcoxon test (Note the highlighted cells in yellow contain tests with significant difference between both the sexes, with *p*-value < 0.05).
(*This table was listed in Excel File:* Microgenderome-OSI-File-C.xlsx)

2. Share species analysis between the male and female

Table S2-1. The results of shared species between the male and female at each of the 15 microbiome sites

	Sample Sites	Observed shared OTUs	Algorithm A1			Algorithm A2		
			Expected shared OTUs	O/E (%)	*p-value*	Expected shared OTUs	O/E (%)	*p-value*
Airway	Anterior nares	8324	9838.246	84.61	<0.001	8528.463	97.60	0.046
Gut	Stool	5806	7193.979	80.71	<0.001	6017.248	96.49	<0.001
Oral	Attached Keratinized gingiva	6802	7686.89	88.49	<0.001	6833.195	99.54	0.235
	Buccal mucosa	9040	9818.42	92.07	<0.001	9061.358	99.76	0.314
	Hard palate	9372	10291.769	91.06	<0.001	9454.56	99.13	0.063
	Palatine Tonsils	10240	11188.39	91.52	<0.001	10291.242	99.50	0.170
	Saliva	9516	10271.771	92.64	<0.001	9446.889	100.73	0.903
	Subgingival plaque	9116	9967.392	91.46	<0.001	9134.805	99.79	0.329
	Supragingival plaque	8665	9307.136	93.10	<0.001	8705.056	99.54	0.142
	Throat	9596	11002.101	87.22	<0.001	9622.037	99.73	0.325
	Tongue dorsum	8374	8967.636	93.38	<0.001	8437.485	99.25	0.060
Skin	Left Antecubital fossa	7476	9927.947	75.30	<0.001	8121.658	92.05	0.001
	Right Antecubital fossa	7632	9930.257	76.86	<0.001	8007.177	95.31	0.008
	Left Retroauricular crease	10113	11722.291	86.27	<0.001	10366.36	97.56	0.03
	Right Retroauricular crease	9159	11052.915	82.87	<0.001	9606.09	95.35	0.002

Table S2-2. The results of shared species of 5 main phyla between the male and female at each of the 15 microbiome sites

				Actinobacteria				
	Sample Sites	Observed shared OTUs	Algorithm A1			Algorithm A2		
			Expected shared OTUs	O/E (%)	*p-value*	Expected shared OTUs	O/E (%)	*p-value*
Airway	Anterior nares	5382	5765.062	93.36	<0.001	5532.263	97.28	0.049
Gut	Stool	28	32.757	85.48	0.015	26.840	104.32	0.796
Oral	Attached Keratinized gingiva	843	1037.582	81.25	<0.001	853.807	98.73	0.281
	Buccal mucosa	1659	1772.427	93.60	<0.001	1642.684	100.99	0.777
	Hard palate	2241	2452.343	91.38	<0.001	2269.059	98.76	0.109
	Palatine Tonsils	2244	2451.098	91.55	<0.001	2223.934	100.90	0.804
	Saliva	1760	1939.827	90.73	<0.001	1748.734	100.64	0.672
	Subgingival plaque	2127	2315.607	91.85	<0.001	2113.833	100.62	0.780
	Supragingival plaque	2149	2313.080	92.91	<0.001	2174.375	98.83	0.038
	Throat	2407	2700.811	89.12	<0.001	2391.171	100.66	0.686
	Tongue dorsum	2497	2630.157	94.94	<0.001	2511.228	99.43	0.186
Skin	Left Antecubital fossa	4156	4804.601	86.50	<0.001	4789.696	86.77	<0.001
	Right Antecubital fossa	4493	5042.368	89.10	<0.001	4906.846	91.57	<0.001
	Left Retroauricular crease	7548	8039.303	93.89	<0.001	7753.422	97.35	0.033
	Right Retroauricular crease	6989	7518.566	92.96	<0.001	7368.900	94.84	0.003
				Bacteroidetes				
	Sample Sites	Observed shared OTUs			Algorithm A1			Algorithm A2
			Expected shared OTUs	O/E (%)	*p-value*	Expected shared OTUs	O/E (%)	*p-value*
Airway	Anterior nares	589	892.128	66.02	<0.001	588.017	100.17	0.489
Gut	Stool	2020	2584.017	78.17	<0.001	2140.717	94.36	<0.001
Oral	Attached Keratinized gingiva	1279	1501.036	85.21	<0.001	1294.023	98.84	0.198
	Buccal mucosa	1767	1947.996	90.71	<0.001	1765.356	100.09	0.525

	Hard palate	1720	1985.719	86.62	<0.001	1744.536	98.59	0.109
	Palatine Tonsils	2373	2633.645	90.10	<0.001	2369.685	100.14	0.572
	Saliva	2393	2614.444	91.53	<0.001	2386.040	100.29	0.628
	Subgingival plaque	2355	2587.205	91.02	<0.001	2381.859	98.87	0.075
	Supragingival plaque	2068	2253.566	91.77	<0.001	2080.072	99.42	0.212
	Throat	1987	2372.735	83.74	<0.001	2011.827	98.77	0.170
	Tongue dorsum	1726	1877.442	91.93	<0.001	1737.669	99.33	0.233
Skin	Left Antecubital fossa	710	1206.133	58.87	<0.001	721.785	98.37	0.298
	Right Antecubital fossa	690	1089.308	63.34	<0.001	669.886	103.00	0.803
	Left Retroauricular crease	498	756.347	65.84	<0.001	501.822	99.24	0.409
	Right Retroauricular crease	389	696.801	55.83	<0.001	392.358	99.14	0.421

Firmicutes

	Sample Sites	Observed shared OTUs	Algorithm A1			Algorithm A2		
			Expected shared OTUs	O/E (%)	*p-value*	Expected shared OTUs	O/E (%)	*p-value*
Airway	Anterior nares	1805	2259.358	79.89	<0.001	1837.939	98.21	0.125
Gut	Stool	3255	3846.363	84.63	<0.001	3339.313	97.48	0.003
Oral	Attached Keratinized gingiva	3096	3293.623	94.00	<0.001	3094.164	100.06	0.540
	Buccal mucosa	3659	3889.352	94.08	<0.001	3685.251	99.29	0.111
	Hard palate	3619	3837.308	94.31	<0.001	3608.788	100.28	0.680
	Palatine Tonsils	3437	3672.194	93.60	<0.001	3472.145	98.99	0.089
	Saliva	2922	3087.149	94.65	<0.001	2890.844	101.08	0.954
	Subgingival plaque	2402	2599.271	92.41	<0.001	2389.725	100.51	0.717
	Supragingival plaque	2257	2382.720	94.72	<0.001	2252.510	100.20	0.631
	Throat	3252	3663.184	88.78	<0.001	3237.996	100.43	0.677
	Tongue dorsum	2408	2545.885	94.58	<0.001	2414.642	99.72	0.334
Skin	Left Antecubital fossa	1898	2547.651	74.50	<0.001	1895.926	100.11	0.472
	Right Antecubital fossa	1771	2387.140	74.19	<0.001	1772.871	99.89	0.436
	Left Retroauricular crease	1626	2095.877	77.58	<0.001	1643.667	98.93	0.236
	Right Retroauricular crease	1411	1857.586	75.96	<0.001	1449.949	97.31	0.086

Fusobacteria

	Sample Sites	Observed shared OTUs	Algorithm A1			Algorithm A2		
			Expected shared OTUs	O/E (%)	*p-value*	Expected shared OTUs	O/E (%)	*p-value*
Airway	Anterior nares	57	84.697	67.30	<0.001	61.271	93.03	0.222
Gut	Stool	2	4.522	44.23	0.039	-	-	-
Oral	Attached Keratinized gingiva	327	370.542	88.25	<0.001	318.838	102.56	0.843
	Buccal mucosa	462	491.775	93.95	<0.001	454.751	101.59	0.854
	Hard palate	388	444.407	87.31	<0.001	401.597	96.61	0.048
	Palatine Tonsils	632	683.097	92.52	<0.001	633.353	99.79	0.448
	Saliva	519	546.095	95.04	<0.001	512.452	101.28	0.788
	Subgingival plaque	690	722.142	95.55	<0.001	682.383	101.12	0.848
	Supragingival plaque	631	673.184	93.73	<0.001	640.385	98.53	0.113
	Throat	498	549.266	90.67	<0.001	507.123	98.20	0.118
	Tongue dorsum	438	495.559	88.39	<0.001	457.431	95.75	0.010
Skin	Left Antecubital fossa	90	141.455	63.62	<0.001	89.137	100.97	0.529
	Right Antecubital fossa	65	107.056	60.72	<0.001	66.774	97.34	0.394
	Left Retroauricular crease	56	96.274	58.17	<0.001	76.107	73.58	0.017
	Right Retroauricular crease	48	75.525	63.56	<0.001	45.328	105.89	0.703

Proteobacteria

	Sample Sites	Observed shared OTUs	Algorithm A1			Algorithm A2		
			Expected shared OTUs	O/E (%)	*p-value*	Expected shared OTUs	O/E (%)	*p-value*
Airway	Anterior nares	412	571.077	72.14	<0.001	414.495	99.40	0.417
Gut	Stool	194	242.133	80.12	<0.001	186.580	103.98	0.928

Oral	Attached Keratinized gingiva	1144	1243.540	92.00	<0.001	1160.327	98.59	0.134
	Buccal mucosa	1252	1358.998	92.13	<0.001	1269.678	98.61	0.096
	Hard palate	1139	1263.493	90.15	<0.001	1166.884	97.61	0.027
	Palatine Tonsils	1116	1236.928	90.22	<0.001	1136.330	98.21	0.086
	Saliva	1392	1496.306	93.03	<0.001	1380.046	100.87	0.785
	Subgingival plaque	1010	1135.579	88.94	<0.001	1035.139	97.57	0.020
	Supragingival plaque	1100	1166.435	94.30	<0.001	1102.189	99.80	0.421
	Throat	1098	1262.996	86.94	<0.001	1111.480	98.79	0.212
	Tongue dorsum	966	1044.546	92.48	<0.001	977.933	98.78	0.138
Skin	Left Antecubital fossa	486	720.529	67.45	<0.001	488.051	99.58	0.422
	Right Antecubital fossa	475	699.964	67.86	<0.001	471.310	100.78	0.611
	Left Retroauricular crease	312	477.992	65.27	<0.001	321.584	97.02	0.175
	Right Retroauricular crease	279	482.396	57.84	<0.001	301.072	92.67	0.036

Table S2-3. The shared and sex-specific OTUs between the male and female at each of the 15 microbiome sites (*This table was listed in Excel File:* Microgenderome-OSI-File-C.xlsx)

3. Sex difference in the inter-individual heterogeneity based on the extended power law

Table S3-1. The parameters of Type-I power law extension (PLE-I) for community spatial heterogeneity and Type-III power law extension (PLE-III) for mixed-species population spatial aggregation (*This table was listed in Excel File:* Microgenderome-OSI-File-C.xlsx)

Table S3-2. The *p*-values of permutation tests for the PLE parameters between the male and female at each of the 15 microbiome sites

	**Sample site**	**Type-I PLE**		**Type-III PLE**	
		b	**ln(*a*)**	***b***	**ln(*a*)**
Airway	Anterior nares	0.983	0.909	0.094	0.112
Gut	Stool	0.731	0.117	0.096	0.033
Oral	Keratinized gingiva	0.160	0.272	0.888	0.884
	Buccal mucosa	0.035	0.031	0.404	0.308
	Hard palate	0.812	0.807	0.733	0.711
	Palatine tonsils	0.441	0.702	0.806	0.705
	Saliva	0.647	0.724	0.991	0.991
	Subgingival plaque	0.360	0.587	0.227	0.194
	Supragingival plaque	0.781	0.977	0.917	0.945
	Throat	0.621	0.753	0.497	0.460
	Tongue dorsum	0.326	0.744	0.841	0.779
Skin	Left antecubital fossa	0.856	0.831	0.006	0.005
	Right antecubital fossa	0.794	0.618	0.013	0.005
	Left retroauricular crease	0.999	0.882	0.507	0.509
	Right retroauricular crease	1.000	0.935	0.009	0.004

4. Sex differences in diversity-scaling profiles with DAR (diversity-area relationship)

Table S4-1. Fitting the DAR models with 100 times of random permutations of samples for the male and female at each of the 15 microbiome sites (*This table was listed in Excel File:* Microgenderome-OSI-File-C.xlsx)

Table S4-2. The p-values of the permutation tests for the differences in the DAR parameters between the male and female at each of the 15 microbiome sites (No parameters exhibited significant differences p=0.05)

Diversity order		Sample site	PL		PLEC			
			z	ln(c)	z	d	ln(c)	*MAD*
	Airway	Anterior nares	0.617	0.659	0.608	0.549	0.654	0.583
	Gut	Stool	0.734	0.758	0.829	0.884	0.813	0.926
		Keratinized gingiva	0.941	0.904	0.938	0.953	0.968	0.456
		Buccal mucosa	0.799	0.908	0.999	0.948	0.979	0.656
		Hard palate	0.958	0.837	0.933	0.926	0.956	0.558
		Palatine tonsils	0.778	0.726	0.993	0.854	0.856	0.987
	Oral	Saliva	0.994	0.852	0.992	0.840	0.838	0.906
q = 0		Subgingival plaque	0.737	0.814	0.806	0.776	0.830	0.927
		Supragingival plaque	0.987	0.981	0.982	0.976	0.974	0.804
		Throat	0.964	0.908	0.972	0.920	0.902	0.851
		Tongue dorsum	0.966	0.909	0.983	0.986	0.982	0.637
		Left antecubital fossa	0.614	0.473	0.680	0.683	0.547	0.421
	Skin	Left retroauricular crease	0.733	0.609	0.835	0.923	0.686	0.563
		Right antecubital fossa	0.638	0.500	0.769	0.854	0.604	0.544
		Right retroauricular crease	0.555	0.406	0.699	0.832	0.505	0.465
	Airway	Anterior nares	0.693	0.535	0.770	0.760	0.620	0.307
	Gut	Stool	0.657	0.647	0.806	0.969	0.709	0.987
		Keratinized gingiva	0.876	0.86	0.942	0.957	0.894	0.286
		Buccal mucosa	0.842	0.852	0.953	0.999	0.914	0.959
		Hard palate	0.902	0.954	0.961	0.960	0.973	0.889
		Palatine tonsils	0.931	0.696	0.972	0.983	0.738	0.312
	Oral	Saliva	0.946	0.739	0.956	0.744	0.792	0.439
q = 1		Subgingival plaque	0.740	0.992	0.749	0.761	0.955	0.246
		Supragingival plaque	0.927	0.766	0.911	0.911	0.829	0.130
		Throat	0.793	0.756	0.988	0.757	0.842	0.797
		Tongue dorsum	0.954	0.984	0.995	0.949	0.989	0.959
		Left antecubital fossa	0.536	0.692	0.548	0.600	0.654	0.704
	Skin	Left retroauricular crease	0.539	0.648	0.651	0.815	0.666	0.781
		Right antecubital fossa	0.586	0.596	0.761	0.909	0.684	0.959
		Right retroauricular crease	0.947	0.747	0.966	0.945	0.818	0.473
	Airway	Anterior nares	0.757	0.533	0.871	0.885	0.646	0.385
	Gut	Stool	0.841	0.753	0.871	0.941	0.777	0.734
		Keratinized gingiva	0.715	0.849	0.765	0.882	0.958	0.286
		Buccal mucosa	0.799	0.888	0.928	0.961	0.940	0.851
		Hard palate	0.955	0.995	0.997	0.981	0.987	0.921
		Palatine tonsils	0.667	0.558	0.913	0.840	0.680	0.746
	Oral	Saliva	0.961	0.795	0.864	0.658	0.904	0.579
q = 2		Subgingival plaque	0.841	0.825	0.772	0.724	0.933	0.280
		Supragingival plaque	0.942	0.794	0.889	0.843	0.820	0.277
		Throat	0.706	0.632	0.817	0.470	0.850	0.411
		Tongue dorsum	0.867	0.948	0.851	0.903	0.883	0.576
		Left antecubital fossa	0.534	0.840	0.571	0.697	0.782	0.440
	Skin	Left retroauricular crease	0.677	0.757	0.676	0.765	0.669	0.855
		Right antecubital fossa	0.625	0.701	0.799	0.978	0.765	0.832
		Right retroauricular crease	0.857	0.923	0.945	0.952	0.923	0.638
q = 3	Airway	Anterior nares	0.722	0.542	0.871	0.885	0.646	0.385
	Gut	Stool	0.997	0.826	0.871	0.941	0.777	0.734
		Keratinized gingiva	0.753	0.852	0.765	0.882	0.958	0.286
		Buccal mucosa	0.848	0.928	0.928	0.961	0.940	0.851
		Hard palate	0.934	0.993	0.997	0.981	0.987	0.921
		Palatine tonsils	0.596	0.557	0.913	0.840	0.680	0.746
	Oral	Saliva	0.960	0.843	0.864	0.658	0.904	0.579
		Subgingival plaque	0.964	0.731	0.772	0.724	0.933	0.280
		Supragingival plaque	0.939	0.782	0.889	0.843	0.820	0.277
		Throat	0.707	0.621	0.817	0.470	0.850	0.411
		Tongue dorsum	0.769	0.912	0.851	0.903	0.883	0.576
	Skin	Left antecubital fossa	0.518	0.869	0.571	0.697	0.782	0.440

	Left retroauricular crease	0.749	0.790	0.676	0.765	0.669	0.855
	Right antecubital fossa	0.548	0.693	0.799	0.978	0.765	0.832
	Right retroauricular crease	0.836	0.960	0.945	0.952	0.923	0.638
	Saliva	0.994	0.852	0.608	0.549	0.654	0.583
	Subgingival plaque	0.737	0.814	0.829	0.884	0.813	0.926
	Supragingival plaque	0.987	0.981	0.938	0.953	0.968	0.456
	Throat	0.964	0.908	0.999	0.948	0.979	0.656
	Tongue dorsum	0.966	0.909	0.933	0.926	0.956	0.558
Skin	Left antecubital fossa	0.614	0.473	0.993	0.854	0.856	0.987
	Left retroauricular crease	0.733	0.609	0.992	0.840	0.838	0.906
	Right antecubital fossa	0.638	0.500	0.806	0.776	0.830	0.927
	Right retroauricular crease	0.555	0.406	0.982	0.976	0.974	0.804

5. Comparing the properties and motifs of basic species co-occurrence networks (SCN)

Table S5-1. The P/N (positive to negative links) ratios of male and female microbial networks at each of the 15 microbiome sites, and the *p*-values of permutation tests for the differences in *P/N* ratios between the male and female

	Sample site	Gender	Positive Links (+)	Negative Links (-)	P/N (+/-) Ratio	*p*-value of permutation test (1000 times of re-sampling)
Airway	Anterior nares	Male	8023	44	182.341	0.602
		Female	5384	2	2692	
Gut	Stool	Male	10936	59	185.356	0.428
		Female	8022	9	891.333	
Oral	Keratinized gingiva	Male	7618	72	105.806	0.868
		Female	5738	40	143.450	
	Buccal mucosa	Male	6142	20	307.100	0.957
		Female	3598	13	276.769	
	Hard palate	Male	5856	236	24.814	0.198
		Female	3038	17	178.706	
	Palatine tonsils	Male	10503	611	17.190	0.072
		Female	5582	37	150.865	
	Saliva	Male	5575	48	116.146	0.818
		Female	1658	9	184.222	
	Subgingival plaque	Male	7119	90	79.100	0.679
		Female	5733	37	154.946	
	Supragingival plaque	Male	8989	116	77.491	0.723
		Female	5707	127	44.937	
	Throat	Male	6730	345	19.507	0.388
		Female	3986	34	117.235	
	Tongue dorsum	Male	18879	2474	7.631	0.167
		Female	9821	567	17.321	
Skin	Left antecubital fossa	Male	4722	10	472.200	0.851
		Female	826	13	63.538	
	Left retroauricular crease	Male	22395	655	34.191	0.687
		Female	24235	348	69.641	
	Right antecubital fossa	Male	4812	9	534.667	0.872
		Female	1784	0	∞	
	Right retroauricular crease	Male	22889	1501	15.249	0.289
		Female	17458	286	61.042	

* The *p*-value of permutation tests for differences of P/N ratio between male and female

Table S5-2A. The occurrences of MAO-trios in the male and female microbial networks, respectively

Microbiome Site		Gender	MAO	Trios with MAO 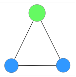					Trios without MAO 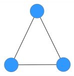				
				+ − − 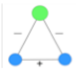	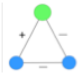	Σ	+ + +	Σ	− − −	+ − −	+ + −	+ + +	Σ
Airway	Anterior nares	Male	*Staphylococcus.43282*	0	0	0	58	58	0	118	0	63292	63410
		Female	*Staphylococcus.43282*	0	0	0	306	306	0	0	0	44241	44241
Gut	Stool	Male	*Bacteroides.38931*	6	27	33	605	638	0	232	0	81108	81340
		Female	*Bacteroides.38931*	0	3	3	476	479	0	8	0	42970	42978
Oral	Keratinized gingiva	Male	*Streptococcus.39439*	79	3	82	17	99	0	147	0	70823	70970
		Female	*Streptococcus.39439*	1	1	2	4	6	0	48	0	59668	59716
	Buccal mucosa	Male	*Streptococcus.39439*	0	0	0	7	7	0	16	0	31605	31621
		Female	*Streptococcus.39439*	0	0	0	9	9	0	10	0	11067	11077

	Hard palate	Male	*Streptococcus.39439*	0	0	0	51	51	0	794	0	29146	29940
		Female	*Streptococcus.39439*	0	0	0	45	45	0	10	0	8166	8176
	Palatine tonsils	Male	*Veillonella.34247*	0	0	0	14	14	0	5507	0	56332	61839
		Female	*Campylobacter.39329*	0	0	0	417	417	0	45	0	20122	20167
	Saliva	Male	*Campylobacter.39329*	0	0	0	357	357	0	101	0	24244	24345
		Female	*Campylobacter.39329*	0	0	0	13	13	0	0	0	2352	2352
	Subgingival plaque	Male	*Streptococcus.39439*	3	12	15	132	147	0	180	0	26337	26517
		Female	*Streptococcus.39439*	3	2	5	78	83	0	43	0	18323	18366
	Supragingival plaque	Male	*Lautropia.20*	0	0	0	753	753	0	434	0	37172	37606
		Female	*Streptococcus.39439*	0	0	0	64	64	0	672	0	18001	18673
	Throat	Male	*Campylobacter.39329*	0	0	0	361	361	0	2558	0	38368	40926
		Female	*Campylobacter.39329*	0	0	0	160	160	0	17	0	12688	12705
	Tongue dorsum	Male	*Veillonella.34247*	1	7	8	4	12	0	56417	0	217086	273503
		Female	*Veillonella.34247*	0	0	0	136	136	0	4662	0	57658	62320
Skin	Left antecubital fossa	Male	*Propionibacterium.43540*	0	0	0	152	152	0	11	0	29295	29306
		Female	*Propionibacterium.43540*	0	0	0	25	25	0	28	0	1552	1580
	Left retroauricular crease	Male	*Staphylococcus.43282*	64	72	136	500	636	0	4806	0	385071	389877
		Female	*Staphylococcus.43282*	1	36	37	1399	1436	0	2291	0	692861	695152
	Right antecubital fossa	Male	*Propionibacterium.43540*	0	0	0	397	397	0	12	0	33067	33079
		Female	*Propionibacterium.43540*	0	0	0	221	221	0	0	0	6974	6974
	Right retroauricular crease	Male	*Propionibacterium.43540*	228	406	634	1203	1837	0	17806	2	484308	502116
		Female	*Staphylococcus.43282*	0	0	0	893	893	0	1952	0	411564	413516

Table S5-2B. The occurrences of MAO-trios with a handle in the male and female microbial networks, respectively

	Microbiome Site	Gender	Single-Link MAO 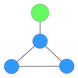			Double-Link MAO 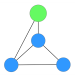				Triple-Link MAO 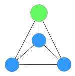				
			−	+	Σ	− −	+ −	+ +	Σ	− − −	+ − −	+ + −	+ + +	Σ
Airway	Anterior nares	Male	0	85	85	0	0	114	114	0	0	0	91	91
		Female	0	169	169	0	0	630	630	0	0	0	1455	1455
Gut	Stool	Male	305	1571	1876	81	48	1362	1491	4	25	92	4133	4254
		Female	64	297	361	0	0	81	81	0	0	3	3136	3139
Oral	Keratinized gingiva	Male	18560	156	18716	3682	5	51	3738	253	0	1	10	264
		Female	2309	26	2335	21	0	10	31	0	0	0	1	1
	Buccal mucosa	Male	0	63	63	0	0	18	18	0	0	0	4	4
		Female	0	23	23	0	0	6	6	0	0	0	7	7
	Hard palate	Male	0	1140	1140	0	0	266	266	0	0	0	55	55
		Female	0	272	272	0	0	192	192	0	0	0	88	88
	Palatine tonsils	Male	0	583	583	0	0	26	26	0	0	0	7	7
		Female	128	4353	4481	0	0	2889	2889	0	0	0	1428	1428
	Saliva	Male	0	7387	7387	0	0	4077	4077	0	0	0	1206	1206
		Female	0	67	67	0	0	4	4	0	0	0	2	2
	Subgingival plaque	Male	278	3498	3776	13	15	1072	1100	0	2	16	263	281
		Female	169	958	1127	15	2	154	171	1	0	0	111	112
	Supragingival plaque	Male	0	1565	1565	0	0	1258	1258	0	0	0	4826	4826
		Female	0	324	324	0	0	102	102	0	0	0	112	112
	Throat	Male	0	12096	12096	0	0	6235	6235	0	0	0	1658	1658
		Female	157	1635	1792	0	0	1094	1094	0	0	0	393	393
	Tongue dorsum	Male	7246	3004	10250	82	198	16	296	0	3	1	0	4
		Female	0	2496	2496	0	0	335	335	0	0	0	218	218
Skin	Left antecubital fossa	Male	0	5732	5732	0	0	1468	1468	0	0	0	373	373
		Female	0	166	166	0	0	45	45	0	0	0	12	12
	Left retroauricular crease	Male	9686	2340	12026	2019	316	1013	3348	164	94	219	2126	2603
		Female	3570	633	4203	34	96	1623	1753	0	10	287	14584	14881
	Right antecubital fossa	Male	0	8961	8961	0	0	4746	4746	0	0	0	1794	1794
		Female	0	1726	1726	0	0	1552	1552	0	0	0	903	903

Right retroauricular crease	Male	14240	40770	55010	3670	3141	12410	19221	1038	1738	3088	11473	17337
	Female	0	1827	1827	0	0	2761	2761	0	0	0	6076	6076

Table S5-3A. The *p*-values of permutation tests for the differences in the numbers of occurrence of MAO-trios between the male and female microbial networks at each of the 15 microbiome sites (with 1000 times of re-sampling)

Treatment		**Trios with MAO** 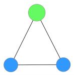					**Trios without MAO** 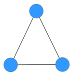				
		+ – – 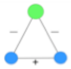	+ – – 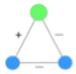	Σ	+ + +	Σ	– – –	+ – –	+ + –	+ + +	Σ
Airway	Anterior nares	1	1	1	1	1	1	1	0.014	0.014	1
Gut	Stool	0.450	0.619	0.499	1	1	1	1	0.191	0.228	1
Oral	Keratinized gingiva	0.676	0.313	0.318	1	1	1	1	0.140	0.301	1
	Buccal mucosa	1	1	1	1	1	1	1	0.782	0.782	1
	Hard palate	1	1	1	1	1	1	1	0.901	0.905	1
	Palatine tonsils	1	1	1	1	1	1	1	0.293	0.305	1
	Saliva	1	1	1	1	1	1	1	0.009	0.009	1
	Subgingival plaque	0.079	1	0.117	1	1	1	1	0.429	0.386	1
	Supragingival plaque	1	1	1	1	1	1	1	0.007	0.007	1
	Throat	1	1	1	1	1	1	1	0.388	0.407	1
	Tongue dorsum	0.211	0.130	0.189	1	1	1	1	0.037	0.069	1
Skin	Left antecubital fossa	1	1	1	1	1	1	1	0.093	0.093	1
	Left retroauricular crease	0.681	0.323	0.468	1	1	1	1	0.002	0.07	1
	Right antecubital fossa	1	1	1	1	1	1	1	0.475	0.475	1
	Right retroauricular crease	0.033	0.087	0.046	1	1	1	1	0.662	0.335	1

Table S5-3B. The *p*-values of permutation tests for the differences in the numbers of occurrences of MAO-trios with a handle between the male and female microbial networks at each of the 15 microbiome sites (with1000 times of re-sampling)

Treatment		**Single-Link MAO** 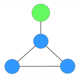			**Double-Link MAO** 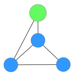				**Triple-Link MAO** 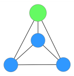				
		–	+	Σ	– –	+ –	+ +	Σ	– – –	+ – –	+ + –	+ + +	Σ
Airway	Anterior nares	1	0.424	0.441	1	1	0.021	0.021	1	1	1	0.017	0.017
Gut	Stool	0.039	0.035	0.015	0.487	0.119	0.015	0.016	0.666	0.545	0.462	0.381	0.381
Ora	Keratinized gingiva	0.174	0.198	0.175	0.305	0.592	0.124	0.305	0.324	1	0.562	0.253	0.326
	Buccal mucosa	1	0.341	0.35	1	1	0.480	0.48	1	1	1	0.465	0.465
	Hard palate	1	0.129	0.152	1	1	0.736	0.737	1	1	1	0.704	0.704
	Palatine tonsils	0.446	0.299	0.307	1	1	0.369	0.377	1	1	1	0.365	0.371
	Saliva	1	0.002	0.002	1	1	0.006	0.006	1	1	1	0.016	0.016
	Subgingival plaque	0.254	0.186	0.184	0.246	0.14	0.161	0.164	0.122	0.084	0.06	0.279	0.261
	Supragingival plaque	1	0.298	0.294	1	1	0.153	0.154	1	1	1	0.005	0.005
	Throat	0.314	0.004	0.004	1	1	0.065	0.064	1	1	1	0.294	0.300
	Tongue dorsum	0.018	0.903	0.295	0.065	0.201	0.442	0.871	1	0.103	0.294	0.107	0.129
Skin	Left antecubital fossa	1	0.073	0.073	1	1	0.037	0.037	1	1	1	0.151	0.151
	Left retroauricular crease	0.66	0.076	0.586	0.389	0.573	0.382	0.548	0.379	0.571	0.853	0.004	0.029
	Right antecubital fossa	1	0.161	0.161	1	1	0.211	0.211	1	1	1	0.584	0.584
	Right retroauricular crease	0.315	0.56	0.419	0.185	0.083	0.586	0.452	0.124	0.069	0.06	0.515	0.312

Table S5-4. Basic network properties of the microbial networks for the male and female, respectively, at each of the 15 microbiome sites

	Treatment	Gender	Num. of Nodes	*Num. of Edges*	*Average Degree*	*Avg. Local Cluster Coefficient*	*Diameter*	*Average Path Length*	*Connected Components*	*Network Density*	*Network Modularity*	*Num. of Communities*
Airway	Anterior nares	Male	630	8068	25.613	0.685	11	3.922	10	0.041	0.795	28
		Female	440	5387	24.486	0.772	13	4.344	8	0.056	0.720	20
Gut	Stool	Male	1115	10996	19.724	0.637	15	5.276	7	0.018	0.773	58
		Female	1082	8032	14.847	0.655	14	5.610	13	0.014	0.865	47
Oral	Keratinized gingiva	Male	731	7691	21.042	0.611	11	4.103	10	0.029	0.626	29
		Female	597	5779	19.360	0.593	15	4.866	4	0.032	0.574	25
	Buccal mucosa	Male	758	6163	16.261	0.553	14	4.599	8	0.021	0.723	38
		Female	695	3612	10.394	0.526	15	5.323	11	0.015	0.791	31
	Hard palate	Male	789	6093	15.445	0.482	12	3.939	9	0.020	0.634	59
		Female	611	3056	10.003	0.521	14	4.699	23	0.016	0.709	68
	Palatine tonsils	Male	1052	11115	21.131	0.483	11	3.958	3	0.020	0.640	98
		Female	847	5620	13.270	0.476	12	4.642	11	0.016	0.669	58
	Saliva	Male	802	5624	14.025	0.460	13	4.122	15	0.018	0.555	58
		Female	616	1668	5.416	0.498	15	5.956	39	0.009	0.796	63
	Subgingival plaque	Male	1159	7210	12.442	0.506	14	4.710	15	0.011	0.750	44
		Female	1069	5771	10.797	0.512	15	5.049	33	0.010	0.751	79
	Supragingival plaque	Male	1146	9106	15.892	0.524	12	4.388	11	0.014	0.721	101
		Female	999	5835	11.682	0.512	13	4.845	11	0.012	0.726	83
	Throat	Male	821	7076	17.238	0.475	11	3.972	10	0.021	0.430	77
		Female	751	4021	10.708	0.526	17	4.818	20	0.014	0.741	40
	Tongue dorsum	Male	1058	21354	40.367	0.516	9	3.008	2	0.038	0.400	146
		Female	887	10389	23.425	0.503	11	3.279	7	0.026	0.567	73
Skin	Left antecubital fossa	Male	452	4733	20.942	0.525	13	3.994	7	0.046	0.548	38
		Female	240	840	7.000	0.550	11	4.796	8	0.029	0.734	19
	Left retroauricular crease	Male	745	23051	61.882	0.579	11	2.796	4	0.083	0.392	51
		Female	588	24584	83.619	0.677	9	2.732	4	0.142	0.225	24
	Right antecubital fossa	Male	416	4822	23.183	0.577	12	3.884	7	0.056	0.445	46
		Female	257	1785	13.891	0.593	11	4.082	9	0.054	0.450	24
	Right retroauricular crease	Male	706	24391	69.096	0.608	10	2.853	2	0.098	0.293	147
		Female	530	17745	66.962	0.684	10	2.759	7	0.127	0.319	24

Table S5-5. The *p*-values of permutation tests for the differences in network properties between the male and female microbial networks at each of the 15 microbiome sites (with 1000 times of re-sampling)

	Treatment	Num. of Nodes	*Num. of Edges*	*Average Degree*	*Avg. Local Cluster Coefficient*	*Diameter*	*Average Path Length*	*Connected Components*	*Network Density*	*Network Modularity*	*Num. of Communities*
Airway	Anterior nares	0.000	0.384	0.929	0.001	0.586	0.668	0.565	0.151	0.242	0.709
Gut	Stool	0.182	0.083	0.029	0.337	0.744	0.177	0.119	0.014	0.040	0.671
Oral	Keratinized gingiva	0.000	0.202	0.652	0.295	0.058	0.053	0.015	0.457	0.362	0.828
	Buccal mucosa	0.000	0.052	0.022	0.178	0.807	0.219	0.495	0.026	0.132	0.662
	Hard palate	0.000	0.049	0.108	0.077	0.511	0.129	0.006	0.286	0.087	0.541
	Palatine tonsils	0.000	0.008	0.018	0.671	0.756	0.021	0.019	0.148	0.611	0.289
	Saliva	0.000	0.012	0.004	0.135	0.688	0.054	0.005	0.001	0.000	0.747
	Subgingival plaque	0.000	0.476	0.534	0.750	0.834	0.486	0.016	0.565	0.983	0.249
	Supragingival plaque	0.000	0.074	0.124	0.541	0.785	0.207	1.000	0.325	0.887	0.448
	Throat	0.001	0.097	0.058	0.010	0.014	0.132	0.023	0.027	0.000	0.181
	Tongue dorsum	0.000	0.014	0.023	0.485	0.329	0.318	0.018	0.053	0.083	0.415
Skin	Left antecubital fossa	0.000	0.248	0.267	0.475	0.604	0.263	0.789	0.485	0.288	0.761
	Left retroauricular crease	0.000	0.804	0.180	0.000	0.236	0.760	1.000	0.001	0.273	0.687
	Right antecubital fossa	0.000	0.213	0.336	0.677	0.843	0.680	0.388	0.929	0.971	0.682
	Right retroauricular crease	0.000	0.416	0.915	0.006	1.000	0.685	0.000	0.212	0.930	0.355

Table S5-6. The strongly connected clusters in the microbial networks of the male and female, respectively, at each of the 15 microbiome sites
(*This table was listed in Excel File:* Microgenderome-OSI-File-C.xlsx)

6. Shared core/periphery network (CPN) analysis between the male and female

Table S6-1. The results of shared core and periphery OTUs between the male and female microbial networks at each of the 15 microbiome sites, based on the *actual-network strategy*.

	Sample Sites	Observed shared OTUs	Algorithm A1			Algorithm A2		
			Expected shared OTUs	O/E (%)	*p-value*	Expected shared OTUs	O/E (%)	*p-value*
**Core**								
Airway	Anterior nares	118	397	29.72	<0.001	392.705	30.05	<0.001
Gut	Stool	217	923	23.51	<0.001	879.419	24.68	<0.001
Oral	Attached Keratinized gingiva	153	500	30.60	<0.001	495.224	30.90	<0.001
	Buccal mucosa	156	581	26.85	<0.001	579.364	26.93	<0.001
	Hard palate	153	571	26.80	<0.001	569.912	26.85	<0.001
	Palatine Tonsils	197	744	26.48	<0.001	740.826	26.59	<0.001
	Saliva	153	532	28.76	<0.001	531.808	28.77	<0.001
	Subgingival plaque	240	880	27.27	<0.001	875.270	27.42	<0.001
	Supragingival plaque	243	837	29.03	<0.001	835.601	29.08	<0.001
	Throat	162	616	26.30	<0.001	608.554	26.62	<0.001
	Tongue dorsum	210	733	28.65	<0.001	732.145	28.68	<0.001
Skin	Left Antecubital fossa	46	294	15.65	<0.001	278.855	16.50	<0.001
	Right Antecubital fossa	58	238	24.37	<0.001	226.565	25.60	<0.001
	Left Retroauricular crease	130	595	21.85	<0.001	576.601	22.55	<0.001
	Right Retroauricular crease	143	478	29.92	<0.001	468.503	30.52	<0.001
**Periphery**								
Airway	Anterior nares	97	454	21.37	<0.001	438.469	22.12	<0.001
Gut	Stool	185	868	21.31	<0.001	831.536	22.25	<0.001
Oral	Attached Keratinized gingiva	142	529	26.84	<0.001	522.736	27.16	<0.001
	Buccal mucosa	130	582	22.34	<0.001	580.222	22.41	<0.001
	Hard palate	113	559	20.21	<0.001	555.969	20.32	<0.001
	Palatine Tonsils	176	778	22.62	<0.001	774.493	22.72	<0.001
	Saliva	144	585	24.62	<0.001	583.895	24.66	<0.001
	Subgingival plaque	201	903	22.26	<0.001	896.902	22.41	<0.001
	Supragingival plaque	202	859	23.52	<0.001	855.728	23.61	<0.001
	Throat	151	639	23.63	<0.001	627.059	24.08	<0.001
	Tongue dorsum	208	790	26.33	<0.001	788.833	26.37	<0.001
Skin	Left Antecubital fossa	41	307	13.36	<0.001	278.500	14.72	<0.001
	Right Antecubital fossa	52	321	16.20	<0.001	294.514	17.66	<0.001
	Left Retroauricular crease	127	477	26.62	<0.001	472.869	26.86	<0.001
	Right Retroauricular crease	105	506	20.75	<0.001	487.652	21.53	<0.001

Table S6-2. The results of permutation tests for shared core or periphery OTUs between the male and female networks at each of 15 microbiome sites, based on the *permutated network strategy* with 1000 times of re-sampling*

	Sample Sites	Core				Periphery			
		O	P	O/P (%)	*p*-value	O	P	O/P (%)	*p*-value
Airways	Anterior nares	118	188.23	62.69	<0.001	97	165.546	58.59	<0.001
Gut	Stool	217	332.213	65.32	<0.001	185	341.658	54.15	<0.001
Oral	Attached Keratinized gingiva	153	208.824	73.27	<0.001	142	223.604	63.51	<0.001
	Buccal mucosa	156	230.581	67.66	<0.001	130	213.952	60.76	<0.001
	Hard palate	153	222.293	68.83	<0.001	113	202.77	55.73	<0.001
	Palatine Tonsils	197	303.828	64.84	<0.001	176	283.564	62.07	<0.001
	Saliva	153	229.019	66.81	<0.001	144	217.786	66.12	<0.001

	Subgingival plaque	240	354.478	67.71	<0.001	201	338.354	59.41	<0.001
	Supragingival plaque	243	335.239	72.49	<0.001	202	313.34	64.47	<0.001
	Throat	162	251.126	64.51	<0.001	151	241.808	62.45	<0.001
	Tongue dorsum	210	301.634	69.62	<0.001	208	289.767	71.78	<0.001
Skin	Left Antecubital fossa	46	138.182	33.29	<0.001	41	136.279	30.09	<0.001
	Right Antecubital fossa	58	132.22	43.87	<0.001	52	122.119	42.58	<0.001
	Left Retroauricular crease	130	219.416	59.25	<0.001	127	207.308	61.26	<0.001
	Right Retroauricular crease	143	217.882	65.63	<0.001	105	204.13	51.44	<0.001

*O: the Observed shared core or periphery nodes; P: the Expected (predicted) shared core or periphery nodes.

Table S6-3. The *p*-values of permutation tests for the differences in core/periphery network (CPN) properties between the male and female with 1000 sets of permutated CPNs (*p*-value<0.05, indicating significant difference)

Microbiome Site		*r*	% of Core	B11	B12 (21)	B22	Nestedness (S)
Airways	Anterior nares	0.000	1.000	0.000	1.000	1.000	1.000
Gut	Stool	0.000	1.000	0.000	0.000	0.000	0.000
Oral	Attached Keratinized gingiva	0.000	0.000	0.000	1.000	1.000	0.000
	Buccal mucosa	0.000	1.000	0.000	0.000	0.000	0.000
	Hard palate	1.000	1.000	1.000	1.000	1.000	1.000
	Palatine Tonsils	0.000	1.000	0.000	0.000	0.000	0.000
	Saliva	0.000	0.000	0.000	0.000	0.000	0.000
	Subgingival plaque	1.000	1.000	1.000	1.000	1.000	0.000
	Supragingival plaque	0.000	0.000	1.000	1.000	1.000	1.000
	Throat	0.000	1.000	1.000	1.000	1.000	0.000
	Tongue dorsum	0.000	0.000	0.000	0.000	0.000	0.000
Skin	Left Antecubital fossa	1.000	0.000	1.000	1.000	1.000	1.000
	Right Antecubital fossa	0.000	0.000	0.000	0.000	0.000	1.000
	Left Retroauricular crease	1.000	1.000	1.000	0.000	0.000	0.000
	Right Retroauricular crease	0.000	1.000	0.000	1.000	1.000	1.000

Table S6-4. The shared and sex-specific core-OTUs between the male and female CPN networks at each of the 15 microbiome sites (*This table was listed in Excel File:* Microgenderome-OSI-File-C.xlsx)

Table S6-5. The shared and sex-specific periphery-OTUs between the male and female CPN networks at each of the 15 microbiome sites (*This table was listed in Excel File:* Microgenderome-OSI-File-C.xlsx)

7. Shared high-salience skeleton network (HSN) analysis between the male and female

Table S7-1. The results of permutation tests for the shared skeletons between the male and female HSN (high-salience skeleton networks) at each of the 15 microbiome sites (with 1000 sets of permutated HSNs)*

Sample Sites		$s > 0$			$s \geq 0.25$			$s \geq 0.5$		
		O	P	*p*-value	O	P	*p*-value	O	P	*p*-value
Airway	Anterior nares	6922	5887.495	0.881	23	49.093	<0.001	2	13.457	<0.001
Gut	Stool	5572	9197.386	<0.001	135	181.947	0.001	23	27.362	0.243
Oral	Attached Keratinized gingiva	3486	4603.718	<0.001	65	105.512	<0.001	12	18.329	0.079
	Buccal mucosa	1762	3013.315	<0.001	109	141.56	0.011	22	26.982	0.198
	Hard palate	3418	3551.444	0.335	80	128.384	<0.001	12	28.503	<0.001
	Palatine Tonsils	4018	5023.687	0.421	102	141.602	<0.001	19	25.297	0.106
	Saliva	4661	3564.192	0.737	79	146.907	<0.001	14	38.901	<0.001
	Subgingival plaque	10097	9981.557	0.561	143	205.041	<0.001	29	41.822	0.029
	Supragingival plaque	3254	5663.946	<0.001	162	187.265	0.056	40	41.813	0.429
	Throat	2649	3673.576	0.429	82	132.299	<0.001	16	28.824	0.003
	Tongue dorsum	5590	7211.521	<0.001	79	87.592	0.212	11	13.696	0.266
Skin	Left Antecubital fossa	611	2795.747	<0.001	18	32.178	0.007	3	7.372	0.061
	Right Antecubital fossa	1379	3540.057	<0.001	10	25.99	0.002	1	9.702	<0.001
	Left Retroauricular crease	10940	14564.573	<0.001	15	25.452	0.028	2	3.131	0.404
	Right Retroauricular crease	9012	15547.731	<0.001	11	22.176	0.017	3	4.296	0.382

*O: Observed shared skeletons; P: Expected (Predicted) shared skeletons; *s*: Salience value.

Table S7-2. The *p*-values from permutation tests for the differences in the HSN (high salience skeleton network) properties between the male and female with 1000 sets of permutated HSNs (*p*-value<0.05, indicating significant difference)

Sample Sites		Links (%)	Max	Skewness	Kurtosis	r_{HSS}
Airway	Anterior nares	0.000	0.000	0.000	0.000	0.000
Gut	Stool	0.000	0.000	0.000	0.000	0.000
Oral	Attached Keratinized gingiva	0.000	0.000	0.000	0.000	0.000
	Buccal mucosa	0.000	0.000	0.000	0.000	0.000
	Hard palate	0.000	0.000	0.000	0.000	0.000
	Palatine Tonsils	0.000	0.000	0.000	0.000	0.000
	Saliva	0.000	0.000	0.000	0.000	0.000
	Subgingival plaque	0.000	0.000	1.000	0.000	0.000
	Supragingival plaque	0.000	0.000	0.000	0.000	0.000
	Throat	0.000	0.000	0.000	0.000	0.000
	Tongue dorsum	0.000	0.000	0.000	0.000	0.000
Skin	Left Antecubital fossa	0.000	0.000	0.000	0.000	0.000
	Right Antecubital fossa	0.000	0.000	0.000	0.000	0.000
	Left Retroauricular crease	0.000	0.000	0.000	0.000	0.000
	Right Retroauricular crease	0.000	0.000	0.000	0.000	0.000
